# Structural and functional studies of *Arabidopsis thaliana* legumain beta reveal isoform specific mechanisms of activation and substrate recognition

**DOI:** 10.1074/jbc.RA120.014478

**Published:** 2020-07-21

**Authors:** Elfriede Dall, Florian B. Zauner, Wai Tuck Soh, Fatih Demir, Sven O. Dahms, Chiara Cabrele, Pitter F. Huesgen, Hans Brandstetter

**Affiliations:** 1 Department of Biosciences, University of Salzburg, Salzburg, Austria; 2 Central Institute for Engineering, Electronics and Analytics, ZEA-3, Forschungszentrum Jülich, Jülich, Germany; 3 CECAD, Medical Faculty and University Hospital, University of Cologne, Cologne, Germany; 4 Institute for Biochemistry, Faculty of Mathematics and Natural Sciences, University of Cologne, Cologne, Germany

**Keywords:** cysteine protease, crystal structure, pH regulation, transpeptidation, structural biology, plant biochemistry, protein stability

## Abstract

The vacuolar cysteine protease legumain plays important functions in seed maturation and plant programmed cell death. Because of their dual protease and ligase activity, plant legumains have become of particular biotechnological interest, *e.g.* for the synthesis of cyclic peptides for drug design or for protein engineering. However, the molecular mechanisms behind their dual protease and ligase activities are still poorly understood, limiting their applications. Here, we present the crystal structure of *Arabidopsis thaliana* legumain isoform β (AtLEGβ) in its zymogen state. Combining structural and biochemical experiments, we show for the first time that plant legumains encode distinct, isoform-specific activation mechanisms. Whereas the autocatalytic activation of isoform γ (AtLEGγ) is controlled by the latency-conferring dimer state, the activation of the monomeric AtLEGβ is concentration independent. Additionally, in AtLEGβ the plant-characteristic two-chain intermediate state is stabilized by hydrophobic rather than ionic interactions, as in AtLEGγ, resulting in significantly different pH stability profiles. The crystal structure of AtLEGβ revealed unrestricted nonprime substrate binding pockets, consistent with the broad substrate specificity, as determined by degradomic assays. Further to its protease activity, we show that AtLEGβ exhibits a true peptide ligase activity. Whereas cleavage-dependent transpeptidase activity has been reported for other plant legumains, AtLEGβ is the first example of a plant legumain capable of linking free termini. The discovery of these isoform-specific differences will allow us to identify and rationally design efficient ligases with application in biotechnology and drug development.

The plant cysteine proteases of the legumain family (C13 family, EC 3.4.22.34) have an important role in processing and maturation of seed storage proteins within the vacuole and, therefore, are also referred to as vacuolar processing enzymes (VPEs) (1). Plant legumains are structurally related to the mammalian caspases and exhibit a strong substrate sequence preference for cleavage after asparagine and, to a lesser extent, aspartate residues (2, 3). Therefore, they are also synonymously referred to as the asparaginyl endopeptidases (AEP). In contrast to mammals, where only one functional legumain isoform is expressed, *Arabidopsis thaliana* contains four genes coding for legumains (α, β, γ, and δ-VPE) and other plants, even up to eight functional variants (4). Plant legumains are expressed primarily in seeds and vegetative organs, consistent with their phylogenetic grouping into two angiosperm clades, the seed type (β-VPE) and nonseed or vegetative type VPEs (α-, γ- and δ-VPE) (5–8). Vegetative legumains are found in lytic vacuoles and have been suggested to play critical roles in plant programmed cell death and may functionally substitute for the caspases, which are absent in plants (9). Seed type legumains like *Arabidopsis thaliana* legumain isoform β (AtLEGβ) play important functions in the processing and maturation of seed storage proteins within storage vacuoles (10, 11). The importance of legumains is especially illustrated in *Arabidopsis* mutant strains missing all four legumain genes (α, β, γ, and δ), which were shown to accumulate aberrantly processed seed storage proteins (12). Importantly, AtLEGβ can compensate for missing vegetative α and γ proteins, further confirming that AtLEGβ is the main player in precursor protein processing in seeds (10). Known substrates of AtLEGβ include the pro12S globulin and pro2S albumin proteins (5, 10, 12, 13).

On top of that, several plant legumains possess peptide ligase and cyclase activity (14–20). Recently, we showed that the vegetative type AtLEGγ harbors ligase activity (21). However, it is still unknown whether this is also true for the other three *A. thaliana* legumain isoforms, especially the phylogenetically more distant seed type AtLEGβ.

Cyclic peptides are important for plant defense against pathogens (16, 17, 22, 23). Well-characterized examples include the kalata B1 peptide, found in *Oldenlandia affinis*, which has proven antimicrobial and insecticidal activities, and the Sunflower trypsin inhibitor 1 (SFTI) (22, 24). Cyclic peptides are very resistant to extremes in pH and temperature, making them ideal scaffolds for biotechnological applications and drug design (25–27). Peptide cyclization in plants is typically catalyzed by legumains. Consequently, there is a high interest in understanding the ligation mechanism, specificity, and efficacy of different plant legumain isoforms. Recent studies led to the discovery of a marker of ligase activity (MLA) and a gatekeeper residue (Cys247, *Oldenlandia affinis* numbering) that allow us to predict ligase activity based on sequence information (20, 28). However, to validate these marker regions, experimental data on ligase activity of different legumain isoforms is indispensable.

Structural analysis of plant legumains showed that they are synthesized as inactive zymogens composed of a caspase-like catalytic domain with AEP activity (AEP domain) and a C-terminal death domain-like prodomain (LSAM domain, legumain stabilization and activity modulation domain) that are connected by an activation peptide (AP) harboring the α6-helix (20, 21, 29, 30). Although this tripartite domain architecture (AEP-AP-LSAM) is conserved in mammalian and plant legumains, the activation process of vegetative-type proAtLEGγ (*Arabidopsis thaliana* prolegumain isoform γ) significantly differs from that of human legumain (31, 32). Importantly, proAtLEGγ is present in an enzymatically latent dimer state that is mediated by AP-LSAM–AP′-LSAM′ interactions and depends on pH and protein concentration (21). Furthermore, we have previously shown that conversion to the active, monomeric AEP form, *i.e.* release of the prodomain, proceeds via a previously unknown two-chain intermediate state. Two-chain AtLEGγ results from cleavage at the N-terminal side of the α6-helix within the AP and is suppressed by high protein concentration, where AtLEGγ dimerization is favored. Even after an initial cleavage within the AP, an enzymatically latent, dimeric two-chain AtLEGγ intermediate form remains stable at neutral pH. Only at acidic pH does the dimer dissociate to monomeric two-chain legumain, which may further release the LSAM domain and thereby convert to the mature AEP form. The identification of the dimer and two-chain states allowed the development of a pH-dependent four-step activation model of plant legumains, *i.e.* single chain–two chain conversion, α6-helix destabilization, dimer–monomer dissociation, and AEP–LSAM release. However, given the subtle regulation of these conversions, isoform-specific differences in activation are to be expected, with experimental data still lacking.

Here, we present the crystal structure of zymogenic proAtLEGβ, which led to the discovery of a distinct activation mechanism, in contrast to AtLEGγ. Combining structural and biochemical information, we show, for the first time, that plant legumains follow isoform-specific autocatalytic activation mechanisms and differential strategies of activity regulation and stability. Furthermore, we provide evidence that seed type AtLEGβ is an active ligase capable of peptide cyclization. AtLEGβ ligase activity is not strictly linked to peptide bond cleavage but enables the efficient joining of free N and C termini. To our knowledge, AtLEGβ is the first example of a plant legumain for which we could demonstrate the ligation of free peptide termini.

This study broadens our understanding of isoform-specific differences in plant legumains and their relevance in plant physiology. Furthermore, the study discloses new avenues to rationally design peptide ligases with applications in biotechnology and drug development.

## Results

### Crystal structure of proAtLEGβ

**Figure 1.**
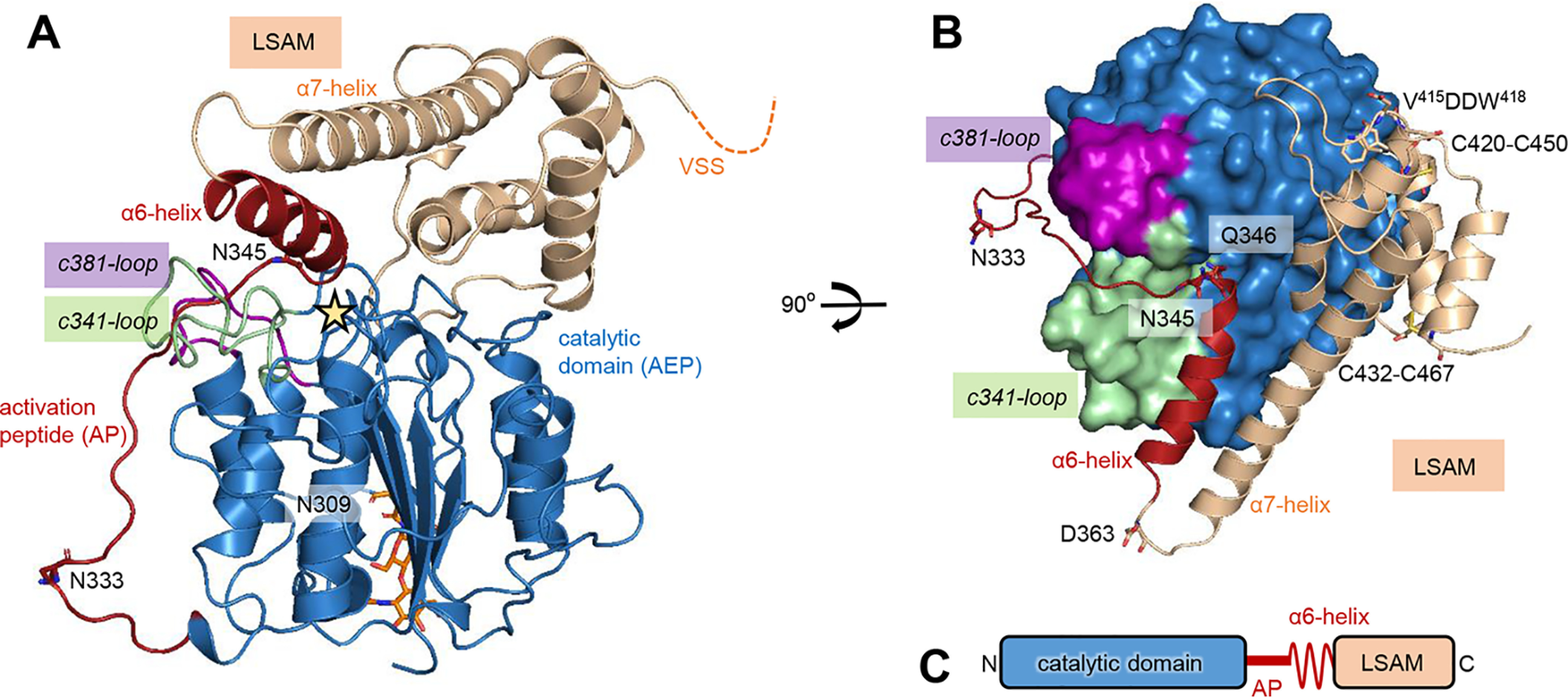
**proAtLEGβ shares the typical prolegumain-like architecture.** *A*, cartoon representation of proAtLEGβ with the catalytic AEP domain shown in *blue*, the activation peptide harboring the α6-helix in *red*, and the LSAM domain in *beige*. Asn333 and 345 autocatalytic cleavage sites and the Asn309 glycosylation site are indicated as *sticks*, an *asterisk* is labeling the active site, and the C-terminal vacuolar sorting signal (VSS) is indicated by a *dashed line*. c341- and c381-specificity loops are colored *green* and *purple*, respectively. *B*, top view of the active site in standard orientation (substrate binding from left to right). Gln346 (*red sticks*) on the AP binds to the S1 pocket. Disulfide bonds on the LSAM domain are shown as *sticks*. The autocatalytic processing sites Asp363 and Asp416 (within the V^415^DDW^418^ motif) are indicated. *C*, schematic representation of proAtLEGβ domain architecture.

**Figure 2.**
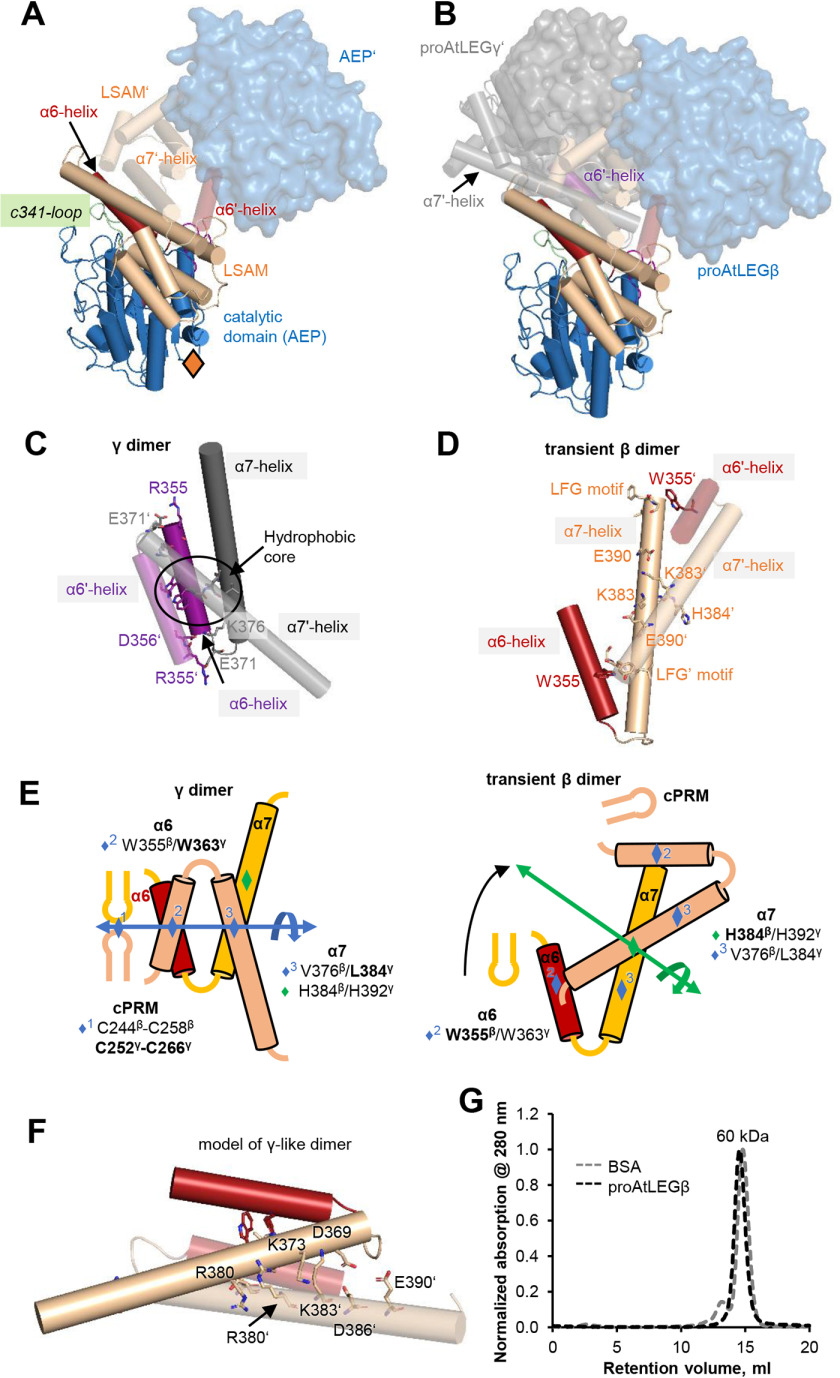
**proAtLEGβ is monomeric in solution.** *A*, crystal packing induced proAtLEGβ dimerization. Monomer 1 is shown in cartoon representation, and monomer 2 is labeled with a prime symbol (AEP′ in surface representation). The location of the Asn309 glycosylation site is indicated with an *orange diamond*. *B*, superposition of *panel A* (proAtLEGβ dimer observed in the crystals) with dimeric two-chain AtLEGγ (PDB entry 5nij). Dimerization led to different spatial orientation of the AEP domains. *C*, zoom-in view on the 4-helix bundle as observed in two-chain AtLEGγ. Interaction is mediated by a hydrophobic core that is surrounded by electrostatic interactions. *D*, zoom-in view on the 4-helix bundle observed in proAtLEGβ. Interaction is mediated by a symmetric E390–K383 salt bridge localized on the α7-helix and hydrophobic interactions between the LFG motif (Leu396-Gly398) on the helix (or α7′-helix) and W355′ on the α6′ helix (or α6 helix). Relative to *panel B*, the views in *panels C* and *D* are rotated by 90° along the *y* axis. *E*, schematic representation of the 4-helix bundle as observed in AtLEGγ and β. *F*, model of an AtLEGγ-like dimerization mode in proAtLEGβ. AtLEGγ-like dimerization is not favored because of electrostatic repulsion of R380–R380′, K373–K383′, and D369–D386′–E390′ pairs. *G*, size exclusion runs confirming monomeric state of proAtLEGβ. BSA served as a size standard.

To understand isoform-specific differences between different AtLEGs, we determined the crystal structure of seed-type proAtLEGβ to a resolution of 2.0 Å (Table 1). The asymmetric unit of the tetragonal space group contained 12 independent molecules. Like isoform γ, proAtLEGβ comprises an N-terminal caspase-like catalytic domain and a C-terminal legumain stabilization and activity modulation (LSAM) domain with death domain-like topology (Fig. 1 and Fig. S1). The AEP and LSAM domain are connected by an activation peptide that harbors the α6-helix. Overall, the structure of proAtLEGβ closely resembles the structure of the homologous two-chain AtLEGγ indicated by a Cα root mean square deviation (RMSD) of 0.49 Å. However, inspecting the individual subdomains unraveled specific differences. Whereas the catalytic AEP domains of AtLEGβ and γ superimpose very well with an overall Cα RMSD of 0.39 Å, we observed bigger differences in the LSAM domains with a Cα RMSD of 0.78 Å (determined with Pymol). This observation is also in agreement with a higher sequence identity of the β and γ catalytic domains (67% identity) compared with the LSAM domains (56% identity). Furthermore, we observed an isoform-specific glycosylation at Asn309, located at the bottom of the enzyme, which is also conserved in human legumain (Fig. 1*A* and 2*A*).

**Table 1:** **X-ray data collection and refinement statistics*^b^***

Parameter	Value(s) for proAtLEGβ (6ysa)
Data collection	
Space group	*P*4_1_
Cell dimensions	
*a* = *b*, *c* (Å)	170.4, 196.5
Resolution (Å)*^a^*	49.6–2.0 (2.04–2.01)
*R*_merge_	0.12 (1.42)
*R*_pim_	0.08 (0.99)
CC(1/2) (%)	0.99 (0.22)
*I*/σ*I*	6.8 (0.7)
Completeness (%)	90.2 (86.3)
Redundancy	2.8 (2.6)
**Refinement**	
Resolution (Å)	49.6–2.0
No. of unique reflections	336,594
*R*_work_/*R*_free_	20.8/21.8
No. atoms	
Protein	39,124
Ligand/ion	763
Water	2254
Overall B-factor (Å^2^)	36.0
RMSD	
Bond length (Å)	0.01
Bond angle (°)	1.15
Ramachandran plot	
No. of outliers (%)	0.0
No. favored (%)	97.9

*^a^*Highest-resolution shell is shown in parentheses.

*^b^*The structure was determined from a single crystal. The resolution cutoff was set by applying the CC1/2 criterion (59).

### proAtLEGβ forms atypical dimers in the crystal and is monomeric in solution

An important feature of proAtLEGγ is that it exists in a latent dimer state in solution, which is mediated by AP-LSAM–AP′-LSAM′ interactions. This dimer controls both the activation and activity of AtLEGγ (21). Similarly, in the crystal structure of proAtLEGβ, we found all twelve independent protomers in the crystallographic asymmetric unit to engage in symmetric dimer contacts, which were mediated by LSAM–LSAM′ interactions (Fig. 2*A*). However, these interactions were mediated by different amino acids and led to an ∼90° tilted orientation of the monomers within the β- and γ-dimer, respectively (Fig. 2, *B* and *C*). Indeed, detailed analyses of the β and γ dimer interfaces revealed significant, isoform-specific differences. The proAtLEGγ dimer is mediated primarily by three symmetric anchoring sites, α6 and α7 helices, and a conserved cyclic protein recognition motif (cPRM) on the c341-loop. The α6 and α7 helices form a 4-helix bundle that is stabilized around a symmetric hydrophobic core formed by W363^γ^ as well as Val383^γ^ and L384^γ^, respectively (AtLEGγ numbering; Fig. 2, *C* and *E*). This hydrophobic core is further stabilized by a network of salt bridges on the N-terminal (R355^γ^–E371^γ^′ and D356^γ^–K376^γ^′) and C-terminal (K376^γ^–D356^γ^′ and E371^γ^–R355^γ^′) ends of the α6-helices. In contrast, the proAtLEGβ dimers in the crystal structure were predominantly mediated by the α7 helix. This interaction was formed around the symmetric H384^β^ (H392^γ^) and further stabilized by one symmetric salt bridge (E390^β^–K383^β^′) as well as by a hydrophobic contact of the α7 C-terminal LFG motif (396^β^-398^β^) with W355^β^′ centered in the α6 helix (Fig. 2, *D* and *E*). The hydrophobic core of the α6-α7, α6′-α7′ four-helix bundle was missing, as was any stabilization by the conserved cPRM, despite key residues important for proAtLEGγ-like dimer formation being conserved in proAtLEGβ (Fig. S1). However, modeling a proAtLEGγ-like dimer uncovered repulsive charge densities of α7-α7′ helix contact residues in AtLEGβ (R380–R380′, K373–K383′, and D369–D386′) that will prohibit this γ-mode of dimerization (Fig. 2*F* and Fig. S2). Together, these findings suggest that the observed β-dimer is weak and probably only transient in solution. To test this conclusion, we performed size exclusion chromatography (SEC) experiments. As expected, at pH 7.0, proAtLEGβ migrated at the expected size of a monomer, similar to human legumain (Fig. 2*F*). Accordingly, proAtLEGβ was a monomer in solution.

### Conserved Gln346 keeps proenzyme in latent state

**Figure 3.**
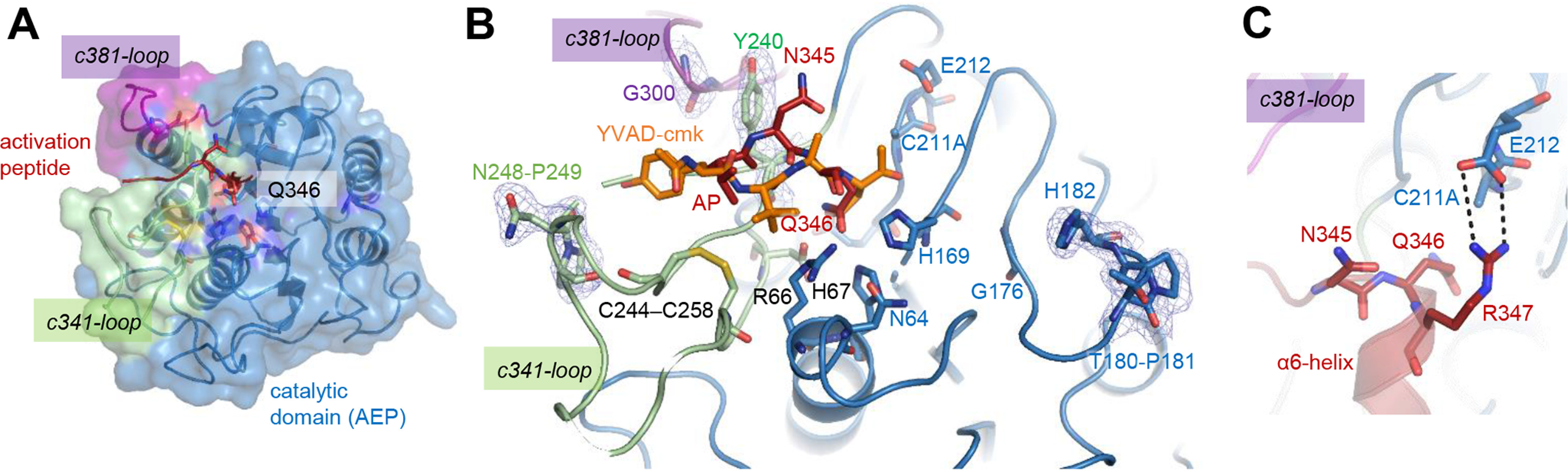
**The activation peptide binds canonically to the active site.** *A*, top view on the active site of proAtLEGβ. The activation peptide (AP) harboring the autocatalytic Asn345 cleavage site and Gln346 that is occupying the S1 pocket are shown in red. *B*, zoom-in view on the nonprime and prime substrate binding sites with a YVAD-cmk peptide modeled based on the crystal structure of the YVAD–AtLEGγ complex (PDB entry 5obt). Cis-imide peptide bonds (Thr180-Pro181 and Asn248-Pro249) are shown as *sticks*. For selected residues, a 2Fo-Fc composite omit map is displayed at a contour level of 1 σ. *C*, zoom-in view on the active site of proAtLEGβ. The ionic clamp (R347–E312) that links the α6-helix to the active site is indicated.

Comparing the crystal structure of proAtLEGβ with YVAD-cmk-inhibited AtLEGγ, we found that the AP binds to the nonprime substrate binding sites in a substrate-like orientation, similar to what we previously observed in mammalian prolegumain (Fig. 1*B* and 3, *A* and *B*). Therefore, the AP is blocking substrate access, keeping the proenzyme in a latent, inactive state. Additionally, we observed a conserved Gln346 (AtLEGβ numbering) on the N-terminal end of the α6 helix. Gln346 is binding into the S1 pocket in an unproductive orientation and thereby preventing cleavage of the AP and further blocking substrate access to the active site (Fig. 1*B* and 3*B*). This interaction was similarly observed in the crystal structure of *A. thaliana* legumain isoform γ; additionally, Gln346 is conserved throughout the plant VPE sequences, strongly suggesting that the Gln346-S1 binding forms a conserved mechanism in plant legumain activity regulation. Additionally, this interaction is further strengthened by the neighboring Arg347, which forms ionic interactions with Glu212, directly next to the catalytic Cys211 (Fig. 3*C*).

Similar to two-chain AtLEGγ and mammalian prolegumains, the LSAM domain is further stabilized by two conserved disulfide bonds (Figs. 1*B* and 3*B*). On the C-terminal end of the LSAM domain, AtLEGβ harbors a potential vacuolar sorting signal, which, however, is not structured and, therefore, not visible in the electron density (Fig. 1*A*).

### Activation proceeds via two-chain intermediate state

**Figure 4.**
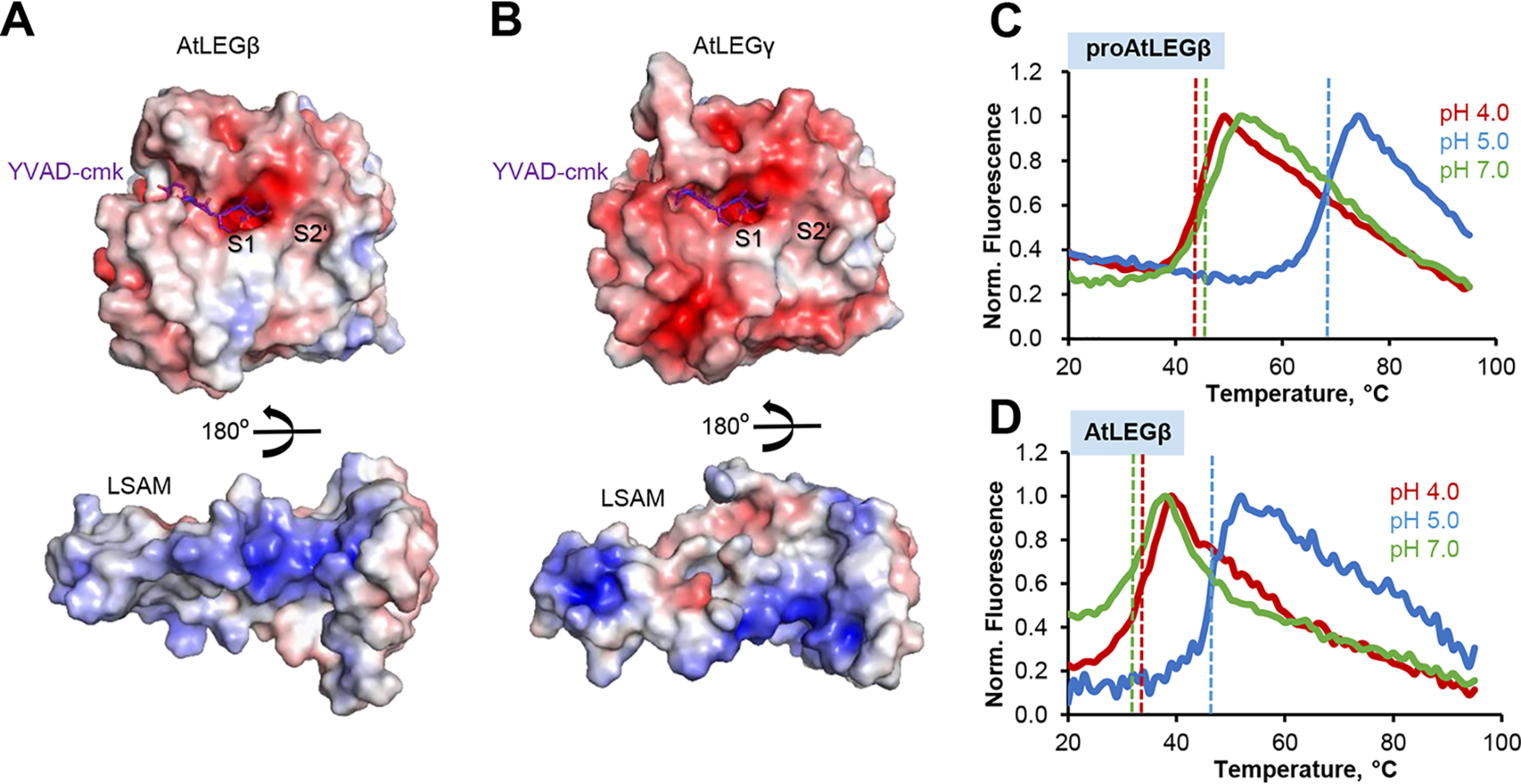
**The AEP**–**LSAM interaction in proAtLEGβ is mostly hydrophobic.** *A*, color-coded electrostatic surface potential of AtLEGβ AEP and LSAM domains based on the crystal structure of proAtLEGβ (*blue*, positive charge, *red*, negative charge) calculated at pH 7.0 and contoured at ±5 kT/e. The LSAM domain has been rotated by 180° relative to the AEP domain. The YVAD-cmk inhibitor has been modeled based on the crystal structure of the AtLEGγ inhibitor complex (PDB entry 5obt). *B*, same as *panel A*, but calculated for AtLEGγ in complex with YVAD-cmk inhibitor. *C*, melting curves of proAtLEGβ at indicated pH values show highest thermal stability at pH 5. Melting points are indicated by *dashed lines*. *D*, melting curves of active AtLEGβ showing highest stability at pH 5.0.

In an effort to unravel the basic principles of proAtLEGβ activation, we analyzed the interdomain interfaces of AEP and LSAM domains. Interestingly, we found that the interface has a hydrophobic character with only two salt bridges identified by PDBe Pisa, R347-E212 and K422-D187, which are also conserved in proAtLEGγ (R355^γ^-E220^γ^ and K432^γ^-D195^γ^; Fig. 4*A*). This is in stark contrast to proAtLEGγ, where the interdomain interface has a mixed charged-hydrophobic character, which is reflected by eight interdomain salt bridges and a hydrophobic cluster localized to the prime substrate binding sites (Fig. 4*B*). Interestingly, the conserved D358^γ^-R74^γ^ (D348^β^-R66^β^) and D358^γ^-H177^γ^ (D348^β^ –H169^β^) form salt bridges in proAtLEGγ, but not in proAtLEGβ, because of a local reorientation of the α6 helix. The residues involved in other AtLEGγ-specific interdomain salt bridges are not conserved in AtLEGβ, *i.e.* K365^γ^-E109^γ^ (M357^β^-L101^β^), R375^γ^-E109^γ^ (K367^β^-L101^β^), R375^γ^-E264^γ^ (K367^β^-I256^β^), and R490^γ^-D136^γ^ (L482^β^-S129^β^). Combined with the differences in oligomerization state, these findings led to the hypothesis that there will be pronounced differences in the activation and pH stability profiles of the two *A. thaliana* legumain isoforms.

**Figure 5.**
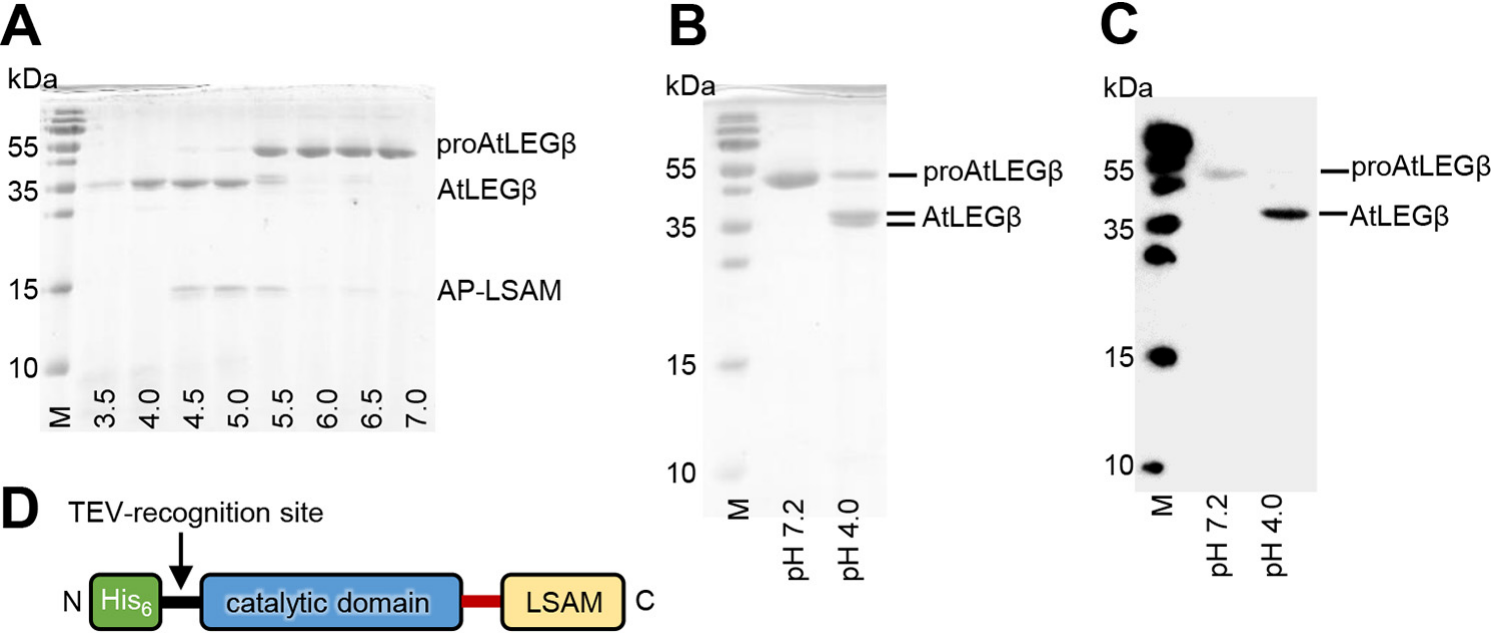
**Autocatalytic activation of AtLEGβ is pH dependent and results in a two-chain intermediate state (pH 5.0) and active AEP state (pH 4.0).** *A*, ProAtLEGβ after 1-h incubation at indicated pH values. AtLEGβ corresponds to the catalytic domain up to the autocatalytic cleavage site Asn333, and AP-LSAM corresponds to the Gln346–Ala486 C-terminal fragment that is generated by cleavage after Asn345. *B*, SDS-PAGE showing proAtLEGβ at pH 7.2 and AtLEGβ following activation at pH 4.0. Activation results in a double band at around 36 kDa. *C*, Western blot using an anti-His-HRP antibody, showing that only one AtLEGβ activation product harbors the N-terminal His_6_-tag. *D*, scheme illustrating the domain architecture of the recombinant expression construct.

Because the interaction between the catalytic domain and the LSAM domain in proAtLEGβ is primarily hydrophobic in nature, we expected that its activation would be rather independent of pH. Surprisingly, an SDS-PAGE-based, pH-dependent activation assay uncovered that the activation profile of AtLEGβ closely resembles that of mammalian legumain, with complete activation only occurring at very acidic pH (4.0) (Fig. 5*A*). Consequently, we hypothesized that autocatalytic activation requires conditions that will destabilize the LSAM domain to gain accessibility to the active site. Indeed, we found complete degradation of the LSAM domain at pH ≤4.0, indirectly indicating that the LSAM domain is destabilized under acidic pH conditions (Fig. 5*A*). Interestingly, upon incubation at pH 5.0, proAtLEGβ was split into catalytic (AEP) and LSAM domains. However, the LSAM domain was not degraded but remained stable on SDS-PAGE. This suggested to us that AtLEGβ forms a two-chain state, where cleavage between LSAM and the catalytic domain occurred but both domains remained bound to each other. To test this, we performed SEC experiments using proAtLEGβ activated at pH 5.0. Indeed, we found a mixture of the two-chain state and isolated AEP domain (Fig. S3). Importantly, there was no dimeric two-chain intermediate state of AtLEGβ observed in SEC after activation.

### Proteolytic activation is initiated by cleavages in the AP

Using MS, we could identify two main autocatalytic cleavage sites, Asn333 and Asn345, on the AP (Fig. 1). These sites were similarly observed in proAtLEGγ and seem to be equally accessible to cleavage. Upon incubation at pH <5.0, we observed additional cleavage sites on the LSAM domain, including Asp363, Asp416, and Asp417 (Fig. 1*B*). Because of the architecture of the S1-pocket, cleavage after Asp is restricted to low-pH conditions (<5.0), in line with the observed cleavage pattern. Interestingly, Asp363 is localized between the α6- and α7-helices and could, in combination with processing at Asn333/345, allow the selective release of the α6-helix (fragment Gln346-Asp363), as observed in mammalian legumain (31). Asp416 and Asp417 are localized within the V^415^DDW^418^ motif, right before the α9-helix (Fig. 1*B* and Fig. S1). This motif is conserved within plant legumains, and cleavage within this sequence was previously shown to be critical for the autocatalytic activation of castor bean legumain (33). Taken together, activation of AtLEGβ at pH <5.0 goes along with cleavage at the aforementioned Asn and Asp sites, which finally results in the complete removal of the AP (including the α6-helix) and the LSAM domain, thereby rendering the active site accessible for substrates.

In addition to cleavage on the AP and LSAM domain, we observed another processing at the N-terminal end of the protein. Here, it is important to note that our proAtLEG expression constructs typically carry an N-terminal His_6_ tag followed by a TEV recognition site (ENLYFQG; TEV, tobacco etch virus protease). We found that AtLEGβ was capable of cleaving after the Asn residue within the TEV recognition site and thereby removing the His_6_ tag, as evidenced by a Western blot using an anti-His antibody (Fig. 5, *B* and *C*). Based on SDS-PAGE experiments, we propose that the primary cleavage at the Asn333/345 cleavage site can be catalyzed by the two-chain form. However, because N-terminal processing within the TEV recognition motif was only observed under very acidic pH conditions, we suggest that the latter cleavage is performed by the fully activated AtLEGβ. Importantly, N-terminal cleavage is not a physiological event, as the relevant sequence is not present in native proAtLEGβ (Fig. S1).

### (pro)AtLEGβ is stable at intermediate pH

Based on the remarkable variances we observed at the AEP–LSAM interfaces of proAtLEGβ and γ, we hypothesized that they would translate into differences of their pH stability profiles. Indeed, when we measured the thermal stability of proAtLEGβ using differential scanning fluorimetry, we found a stability optimum at pH 5.0 (Fig. 4*C*). This is very different from proAtLEGγ and mammalian legumain, where the stability optimum of the proenzyme is at neutral pH (21, 32). Even more interestingly, we found that AtLEGβ activated at pH 4.0 and, thereby, lacking the LSAM domain, similarly showed a maximum in pH stability at pH 5.0 (Fig. 4*D*). This is in stark contrast to AtLEGγ and also mammalian legumain, where the AEP domain is most stable at pH ∼4. However, this difference becomes clear considering the hydrophobic interaction between AEP and LSAM domain in proAtLEGβ. Mammalian legumain and AtLEGγ harbor a highly charged electrostatic stability switch (ESS) on the AEP surface, located at the area surrounding the active site (32). At neutral pH conditions, the ESS causes electrostatic destabilization of the isolated AEP domain because of the high negative-charge density, which is not compensated for by the LSAM domain. In human legumain and AtLEGγ, the isolated AEP can be stabilized by protonation of the excess acidic residues, hence the maximum stability at pH 4. The AEP in AtLEGβ lacks the pronounced ESS, explaining why a strong acidic pH is not necessary for charge neutralization, in agreement with the pH optimum at 5.0. The interaction of the AEP with LSAM generally stabilizes the protein. In proAtLEGβ, AEP–LSAM interaction and stabilization do not depend on neutral pH, whereas the tight electrostatic clamping of these domains in proAtLEGγ and human prolegumain depend on neutral pH. Consequently, proAtLEGβ is most stable at the pH that is also favorable for the isolated AEPβ.

### Overall topology of AEP domain is highly conserved

**Figure 6.**
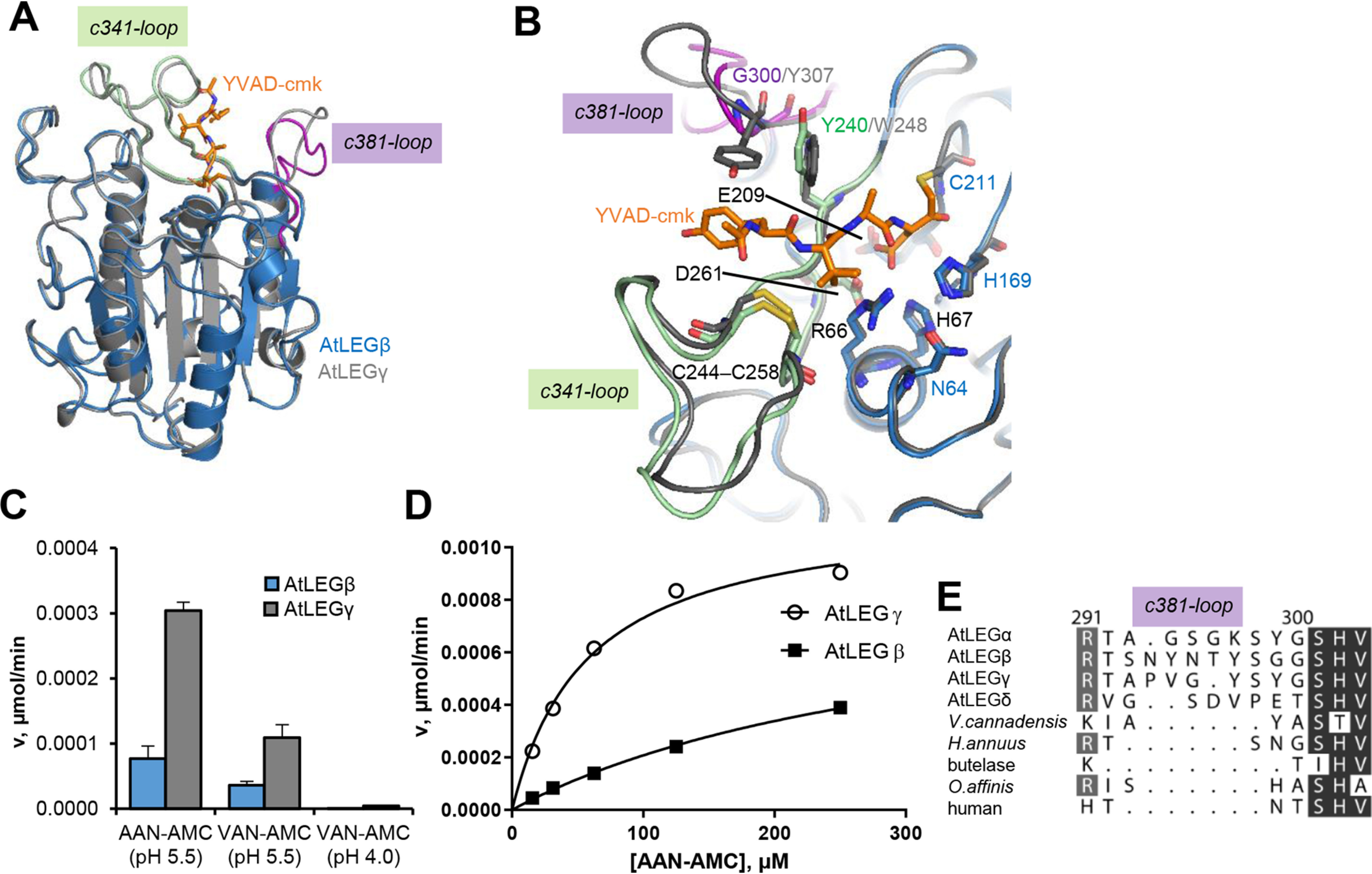
**AtLEGs differ in their substrate specificity loops.** *A*, superposition of AtLEGβ (*blue*) and γ (gray) AEP domains. The YVAD-cmk inhibitor bound to AtLEGγ is shown in *orange sticks*, the c341-loop in *green*, and the c381-loop in *purple*. *B*, zoom-in view on the active site. Catalytic residues are labeled in *blue*, and residues forming the S1 specificity pocket are labeled in *black*. *C*, catalytic activities of AtLEGβ and –γ toward peptidic AAN-AMC and VAN-AMC substrates at indicated pH values. *D*, *K_m_* determination for AtLEGβ and –γ toward the AAN-AMC substrate. *E*, sequence alignment of the c381-loops of indicated (plant) species. Sequences were derived from structures deposited in the PDB, where applicable. AtLEGα (P49047), AtLEGβ (Q39044), AtLEGγ (5nij), AtLEGδ (Q9LJX8), *V. canadensis* (*Viola Canadensis*; 5zbi), *H. annuus* (*Helianthus annuus*; 6azt), butelase (*Clitoria ternatea*; 6dhi), and *O. affinis* (*Oldenlandia affinis*; 5hoi) were used.

Previous studies showed that the AEP domain in prolegumain is present already in an active conformation (21, 34). Zymogenicity resulted solely from the steric blockage of the active site by the AP and LSAM domain. Therefore, we can use the crystal structure of proAtLEGβ to analyze the active AtLEGβ state. When we superimposed the AEP domains of AtLEGβ and γ, we found that their fold is highly conserved (Fig. 6*A*). AtLEGβ exhibits a caspase-like topology, *i.e.* a 6-stranded central β-sheet that is surrounded by 5 major α-helices (Fig. S1 and S4) (35). Furthermore, AtLEGβ harbors the c341- and c381-loops, which form the nonprime substrate binding sites. The c341-loop encodes a plant VPE-specific disulfide bond that is stabilizing the proline-rich insertion that is extending the c341-loop compared with mammalian legumain (Fig. 6*B*). Mutation of Cys244 or Cys258 resulted in a complete loss of protein expression, confirming that the disulfide is also critical for folding. Furthermore, we observed 2 cis-imide peptide bonds (Thr180-Pro181 and Asn248-Pro249) with relevance for stable bend and turn formation (Fig. 3*B*) (36). Interestingly, both turns are located in the substrate binding sites. The Asn248-Pro249 cis-peptide bond is on the c341-loop (nonprime side) and presents the Asn248 carbonyl oxygen as the main-chain recognition site for the P4 amide. Thr180-Pro181 is part of the eastern rim of the S2′ pocket.

### AtLEGβ has a wide S3-S4 pocket

When looking into the active site, we found that the active-site residues Cys211, His168, and Asn64 also superimpose very well with the related AtLEGγ (Fig. 6*B*). Furthermore, the residues forming the S1-specificity pocket, Arg66, His67, E209, and D261, adopt conformations identical to those observed in AtLEGγ. The highly conserved architecture of the active site suggested similar substrate specificity and catalytic activity of AtLEGβ and γ. However, when we compared the catalytic activity toward the fluorogenic Ala-Ala-Asn-AMC substrate, we observed a surprisingly low catalytic activity for AtLEGβ compared with γ (Fig. 6*C*). Because the positioning of the active-site residues were basically identical in β and γ, we did not expect this difference in activity to originate from a *k*_cat_ effect but rather from differences in substrate affinity (*K_m_*). Beyond the highly similar S1 pocket, we identified major differences on the c341- and c381-loops on the nonprime side (Fig. 6*B* and Fig. S5). Variations in sequence and conformation resulted in a narrow S3–S4 pocket in AtLEGγ but a rather wide pocket in AtLEGβ (Fig. 4, *A* and *B*, 6, and Fig. S5). To test whether these differences were a result of the induced fit of the YVAD-cmk inhibitor, we superposed the crystal structures of proAtLEGβ, two-chain (pro)AtLEGγ, and active YVAD-AtLEGγ and compared their active sites. Interestingly, we found that the conformations of the substrate specificity loops c341 and c381 of proAtLEGβ most closely resembled the active state of AtLEGγ. Therefore, we could exclude that induced fit was a main regulator of substrate affinity (Fig. S5). However, the situation might be different in AtLEGγ, where we observed pronounced conformational changes of the c381-loop between the proenzyme and the YVAD-cmk inhibited form. Modeling a peptidic substrate, based on the YVAD-cmk-AtLEGγ crystal structure, we found tight interactions in AtLEGγ but fewer interactions to AtLEGβ. Whereas AtLEGβ offered an open, broad surface to accommodate the YVAD substrate, AtLEGγ was tightly embracing the peptidic substrate, as visible in Fig. 4, *A* and *B*, and 6*B*. We could assign Tyr240^β^/Trp248^γ^ on the c341-loop and Gly300^β^/Tyr307^γ^ on the c381-loop as the main determinants for this difference. Together, this suggested to us that small peptidic substrates would bind with lower affinity to AtLEGβ compared with gamma because of missing enzyme-substrate interactions. Indeed, when we determined *K_m_* values for AtLEGβ and γ toward the AAN-AMC substrate, we found high-affinity binding (*K_m_* = 57 ± 3 μm) to AtLEGγ but low affinity for AtLEGβ (*K_m_* = 337 ± 3 μm) (Fig. 6*D*). Importantly, we found similar *k*_cat_ (AtLEGβ, 4.5 × 10^−3^ min^−1^; AtLEGγ, 6.3 × 10^−3^ min^−1^) and *V*_max_ values (AtLEGβ, 0.9 × 10^−3^ µmol/min; AtLEGγ, 1.1 × 10^−3^ µmol/min) for both enzymes. These findings confirmed that the difference in catalytic activity between AtLEGβ and γ was explained by differences in substrate affinity. Interestingly, when we used a VAN-AMC substrate instead of AAN-AMC, we observed a reduction in enzymatic activity for both AtLEGβ and γ (Fig. 6*C*). Accordingly, the smaller alanine is preferred over the branched valine at the P3 position in both AtLEG isoforms. Furthermore, we found an activity optimum for AAN-AMC turnover at pH 5.5, which is also in agreement with the pH stability requirements of the AEP domain (Fig. S6).

### c381-loop is variable in length and sequence

Together, these observations made us hypothesize that the c341- and c381-loops serve as a *K_m_* switch. To analyze this further, we superposed all plant legumain structures available in the PDB. Whereas the main structural elements superimposed very well in all available structures, we observed big differences on the c381-loops. It is variable in length and sequence and may even contain a glycosylation site (Fig. 6*E* and Fig. S4). Together, these findings suggested that the c381-loop is a main determinant of the proteolytic activity of legumains, similar to caspases. The relevance of the c381-loop for legumain activity is further supported by a previous analysis suggesting it as a marker of ligase activity (MLA) (28).

### AtLEGβ substrate specificity is pH dependent

**Figure 7.**
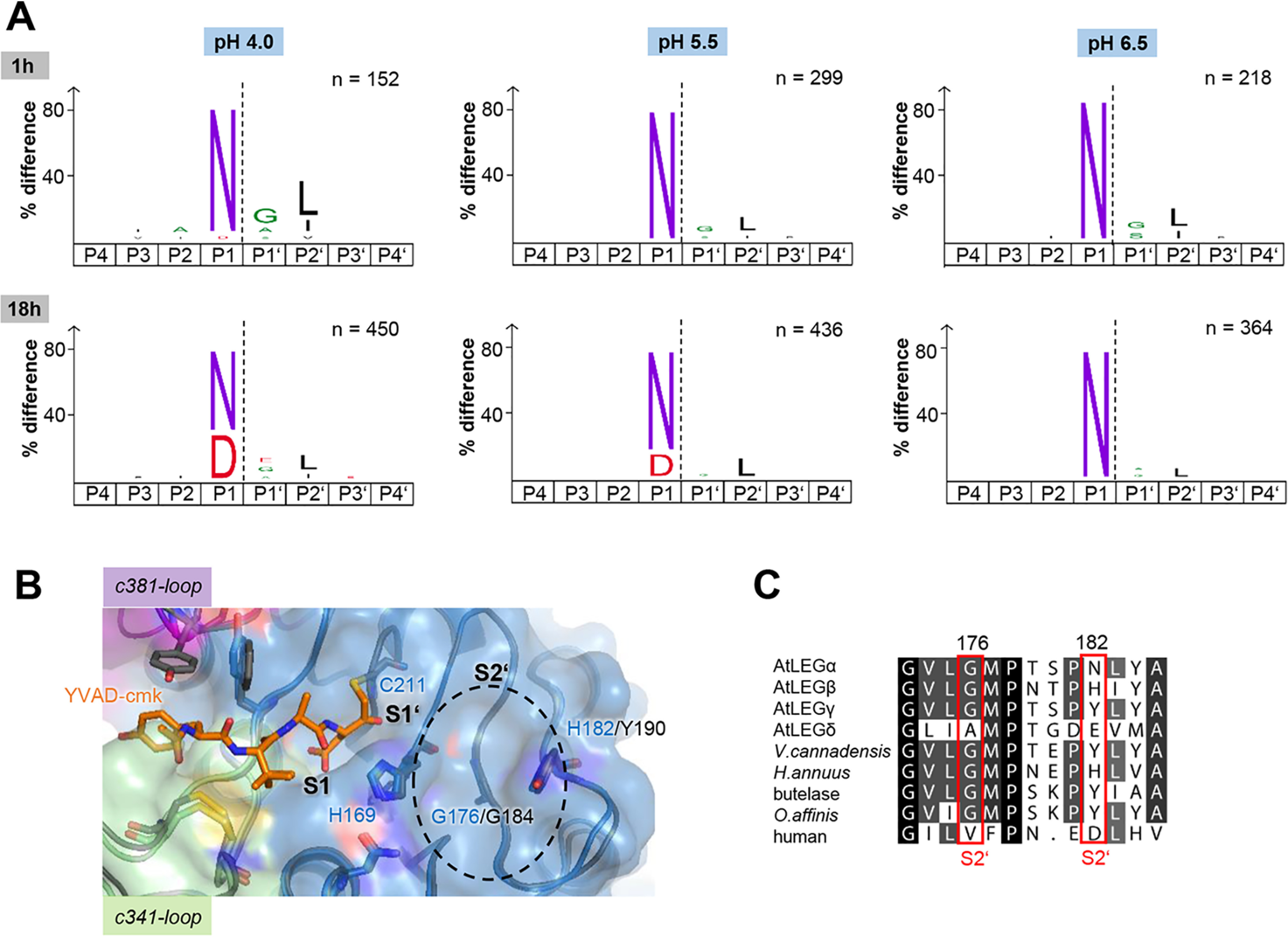
**AtLEGβ has a pH-dependent substrate specificity.** *A*, cleavage site specificity determined by the PICS assay, using peptides generated by tryptic digest of an *E. coli* proteome as the substrate library. iceLogos visualize the substrate preference surrounding the cleavage sites (*p* = 0.05) based on peptides cleaved by AtLEGβ after incubation at indicated pH values and times. The numbers of nonredundant cleavage sites used to generate the iceLogos are indicated. *B*, top view of the AtLEGβ substrate binding site. Binding of the YVAD-cmk inhibitor was modeled based on the crystal structure of the YVAD–AtLEGγ complex (PDB entry 5obt). *C*, sequence alignment of the residues forming the prime-substrate binding site. Sequences used are the same as those in Fig. 6.

To further analyze the substrate specificity of AtLEGβ, we carried out PICS experiments, which use proteome-derived peptides as substrate libraries (37, 38). Here, we used a peptide library that was generated from an *E. coli* proteome by digestion with trypsin for AtLEG specificity profiling under three different pH conditions. As expected, we observed a strong preference for Asn in the P1 position at all investigated pH values (Fig. 7*A*). Interestingly, we also observed an increasing frequency of cleavage at Asp residues upon prolonged incubation times (18 h). This time dependence illustrates that substrates with Asn in P1 are kinetically favored over Asp. The substrate preference was also pH dependent, *i.e.* the turnover rate of P1-Asp substrates increased with lower pH values, which nicely agrees with the bipolar architecture of the S1 specificity pocket and with previously published data for human legumain (Fig. 6*B*) (32).

### AtLEGβ has a strong preference for hydrophobic residues in P2′

Furthermore, we observed a slight preference for small, polar residues in the P1′ position, which was especially visible at the shorter incubation times (Fig. 7*A*), suggesting that P1′-Gly is kinetically preferred. Additionally, we found a pronounced preference for Leu in the P2′ position. Leucine has previously been proven to be beneficial at the P2′ position in legumain ligase substrates (16). Together, these results are in nice agreement with the architecture of the S1′ and S2′ binding sites. Whereas the S1′ binding site is flat and not allowing much interaction with the enzyme, the S2′-binding site forms a pronounced pocket (Fig. 4*A*) (29). Small residues in the P1′ position will facilitate the simultaneous binding of the P1 and P2′ residues into the respective S1 and S2′ binding pockets while still maintaining enough flexibility to allow efficient cleavage of the scissile peptide bond. The bottom of the S2′ pocket is formed by Gly176 and the eastern wall by His182 (Fig. 7*B*). Gly176 is conserved in all plant legumains that have been structurally characterized so far (Fig. 7*C*). The eastern wall is mostly histidine and tyrosine, with some exceptions. Interestingly, mammalian legumain harbors a valine at position 176, making the S2′-pocket shallower and, thereby, less specific at this position (39). Furthermore, AtLEGδ has the glycine replaced by alanine (Fig. 7*C*), suggesting that it also will have a less pronounced specificity at the P2′ position. To test the relevance of His182 for prime side substrate specificity, we repeated the PICS experiments using AtLEGγ, which has a tyrosine at the equivalent position (Fig. S7). Interestingly, we found highly similar preferences on the nonprime and prime substrate binding sites, further confirming that Gly176 is the main determinant at the S2′ site.

### AtLEGβ has a strong preference for small residues in P1′ position in protein substrates

**Figure 8.**
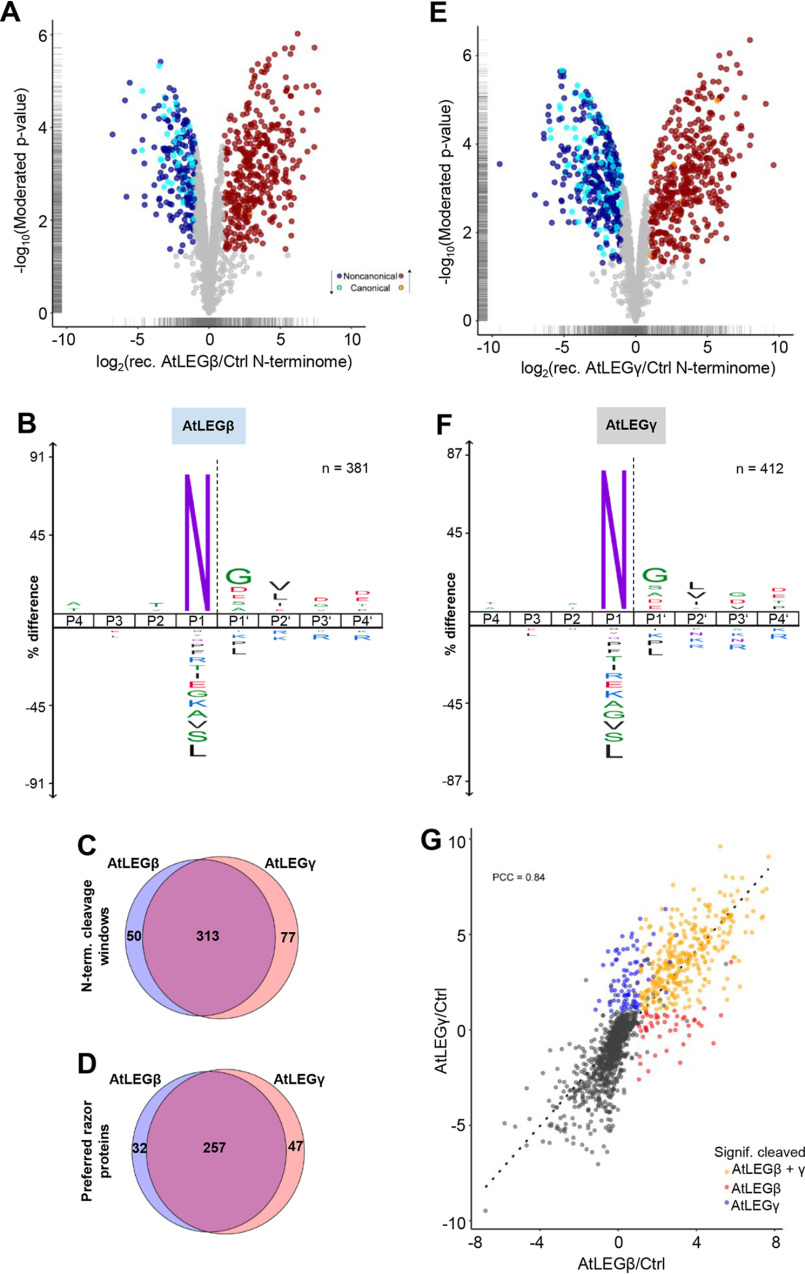
**Substrate specificity of AtLEGβ and** γ **toward intact proteins extracted from *A. thaliana* leaves.** Volcano plots identify protein N-terminal peptides significantly changing in abundance (greater than 2-fold change in abundance supported by LIMMA-moderated *t* test; *p* < 0.05) after *in vitro* incubation of *A. thaliana vpe0* proteome with recombinant AtLEGβ (*A*) or AtLEGγ (*E*). Log_2_ fold change is the mean of 3 biological replicates. Accumulating N-terminal peptides indicative of AtLEGβ/γ cleavage are highlighted *red*, and depleted peptides cleaved within their sequence are in *blue*. iceLogos visualize the substrate preference surrounding the cleavage sites for AtLEGβ (*B*) and AtLEGγ (*F*) (*p* = 0.05). Numbers of nonredundant cleavage sites used to generate the iceLogos are indicated. Venn diagrams show the overlap of cleavage sites (*C*) and proteins (*D*) cleaved in the *vpe0* proteome after incubation with AtLEGβ or γ. *G*, correlation of N-terminal peptide abundance in both experiments (dimethylated N-terminal peptides quantified in at least 2 out of 3 replicates). Significantly accumulating dimethylated N-terminal peptides (log_2_ fold change of >1, LIMMA-moderated *t* test *p* value of <0.05) indicate cleavage by AtLEGβ (*red*), AtLEGγ (*blue*), or both (*orange*). The linear fit confers a Pearson correlation coefficient (PCC) of 0.84, indicating a very high degree of overlap among the putative substrates.

In the next step, we analyzed the substrate specificity of AtLEGβ toward protein substrates, using proteome extracts isolated under nondenaturing conditions from leaves of the *A. thaliana vpe0* mutant lacking expression of all four VPE isoforms as a substrate library. After incubation with recombinant AtLEGβ, recombinant AtLEGγ, or buffer control, free N-terminal α-amines where labeled with three different formaldehyde isotopologues, and cleavage sites were determined using the HUNTER N-termini enrichment and MS (40). Based on three biological replicates, we identified 381 N-terminal peptides significantly accumulating after incubation with AtLEGβ at pH 6.0 (Fig. 8, *A* and *B*, and Table S1), matching to 363 unique cleavage sites (Fig. 8*C*) in 289 proteins (Fig. 8*D*). As expected, we found a pronounced preference for Asn at the P1 position (Fig. 8*B*). Furthermore, we observed a stronger preference for small and polar residues in the P1′ position, suggesting that the accessibility of the scissile peptide bond is enhanced when it is flanked by a small residue. Additionally, we also noticed a slightly increased preference for the more bulky and charged Asp and Glu amino acids. As in the peptide-based PICS experiment, we again observed a preference for hydrophobic amino acids in the P2′ position. For AtLEGγ, we identified 412 significantly accumulating N-terminal peptides (Fig. 8*E*, Table S1). These matched 390 unique cleavage sites (Fig. 8*C*) in 304 proteins (Fig. 8*D*) that exhibited a very similar cleavage profile, in line with our observations using peptide substrates (Fig. 8*F*). Notably, the vast majority of 313 of the cleavage sites in 257 proteins were cut by both enzymes, although only 50 cleavages in 32 proteins were strongly preferred substrates of AtLEGβ and 77 cleavages sites of 47 proteins were selectively cut by AtLEGγ (Fig. 8, *C*, *D*, and *G*).

### AtLEGβ is a broad-spectrum transpeptidase

**Figure 9.**
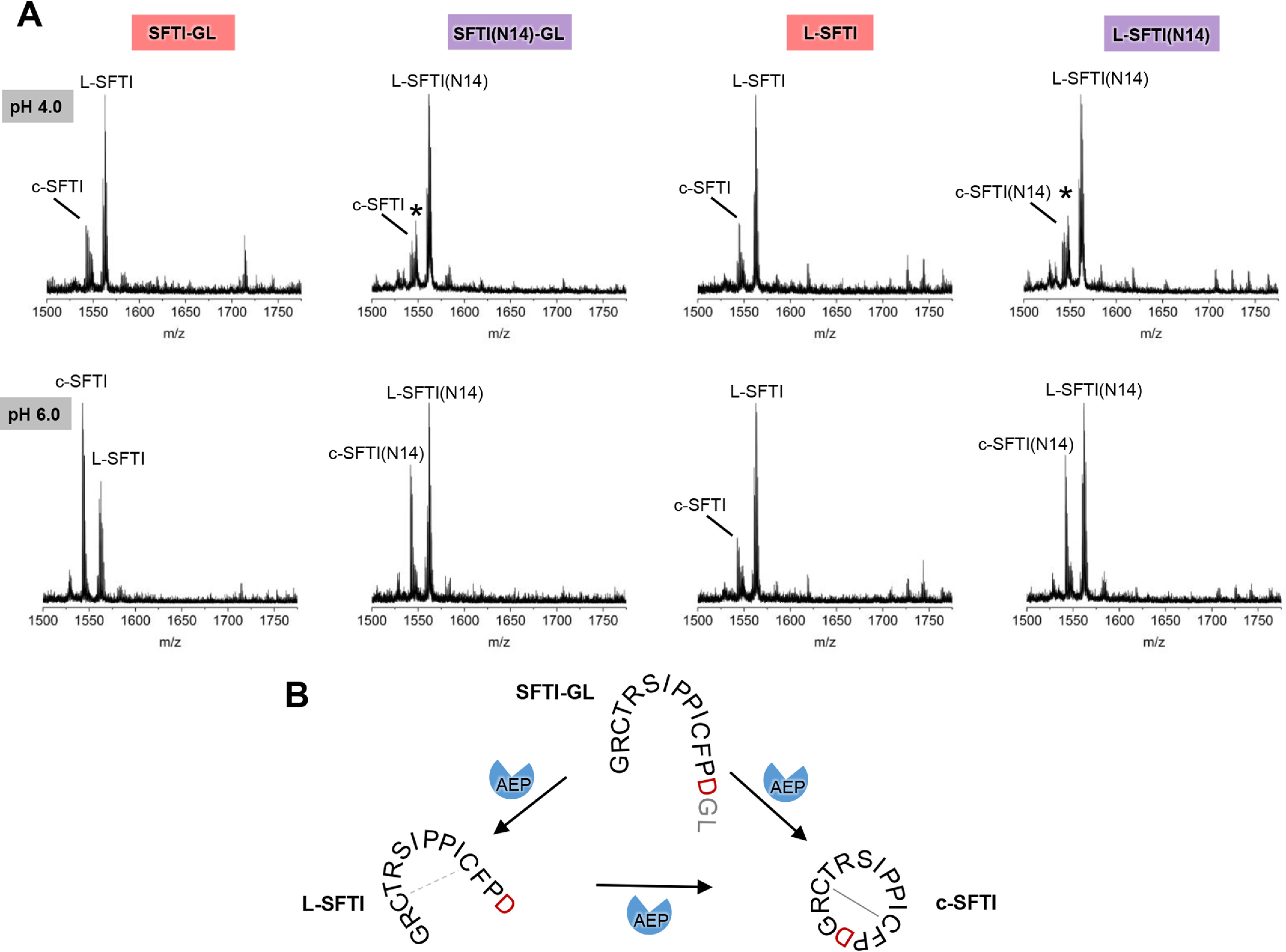
**Cyclization of SFTI-derived peptides by AtLEGβ is pH dependent.** *A*, reactions were carried out at indicated pH values. An unidentified species is labeled with an *asterisk*. *B*, reaction scheme of AtLEGβ catalyzed cyclization of SFTI-GL peptides. The precursor peptides SFTI-GL and SFTI(N14)-GL were synthesized in the reduced form and were also observed mostly reduced in the assays. The linear l-SFTI and l-SFTI(N14) cleavage products were observed both in the reduced and oxidized forms, with the Cys3–Cys11 disulfide bond formed. c-SFTI and c-SFTI(N14) were mostly oxidized.

To characterize the cyclase activity of AtLEGβ, we coincubated it with different SFTI-derived linear peptides and measured the formation of the cyclic product using MS. Indeed, we found that AtLEGβ could cleave the SFTI-GL precursor peptide to the linear l-SFTI (lacking GL) version and further cyclize it to cyclic SFTI (c-SFTI) (Fig. 9*A*). Cyclization worked most efficiently at pH 6.0, which is in agreement with the previously reported pH requirements of legumain ligase activity (29, 41). Using the SFTI-GL precursor peptide, which harbors an Asp at the P1 position, we observed a product formation rate of about 60%. This is less than that with AtLEGγ, which resulted in approximately 80% product formation (29). Interestingly, when the P1 residue was replaced by Asn, as is the case in SFTI(N14)-GL, AtLEGβ was still able to catalyze peptide cyclization, in contrast to the situation of AtLEGγ. When we replaced the P1′-P2′ Gly-Leu with His-Val residues, which is the preferred sequence found for butelase-1 (*C. ternatea* legumain), we observed a similar cyclization efficiency (Fig. S8) (16), showing us that albeit optimized for butelase-1, the HV-dipeptide is not facilitating peptide ligation in AtLEGβ.

### AtLEGβ is a broad-spectrum ligase

Along these lines, we also tested whether AtLEGβ would be able to cyclize linear l-SFTI and l-SFTI(N14) peptides, which lack amino acids on P1′ and P2′ positions of the protease substrate. Surprisingly, AtLEGβ was indeed able to join the free termini and form the cyclic product, suggesting that AtLEGβ is not only a transpeptidase but also a real ligase (Fig. 9, *A* and *B*). Using the SFTI peptides carrying Asn at P1 position (N14), cyclization worked equally well with or without the preceding cleavage of prime side residues. In the case of Asp at the P1 position, transpeptidation (cleavage-linked ligation) was preferred to joining free ends. Again, product formation was pH dependent, working best at near-neutral pH conditions. So far, there was not a single report of a (plant) legumain capable of efficiently linking free peptide termini.

## Discussion

**Figure 10.**
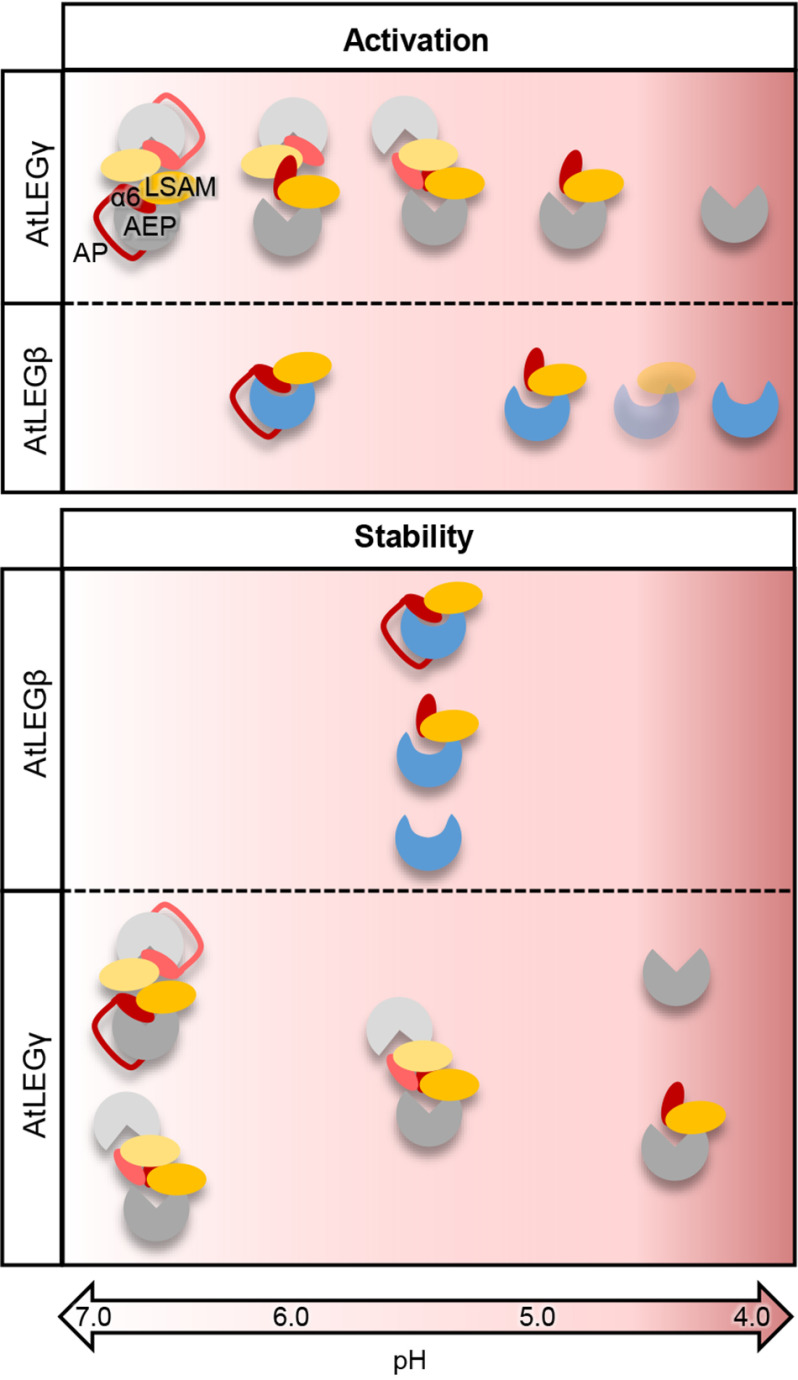
**Activation and stability of AtLEGβ and AtLEGγ are pH dependent.** In the zymogen forms of proAtLEGβ and γ, the LSAM domain (*orange*) and activation peptide (AP, *red line*) that harbors the α6-helix (*red ellipsoid*) sit on top of the active site and thereby block access to the substrate binding sites. ProAtLEGγ forms a dimer at neutral to intermediate pH conditions and is mostly present in its two-chain state, which is generated upon cleavage at the N-terminal end of the α6-helix. By lowering pH, the interaction of the α6-helix with the AEP domain gets weaker, as it is mainly mediated by electrostatic interactions. At pH <4.5, the two-chain state will disassemble and thereby allow degradation of the α6-helix and the LSAM domain. In contrast, proAtLEGβ is a monomer in solution. Activation proceeds via cleavage after (1) Asn333/345 on the N-terminal end of the α6-helix at intermediate pH, followed by (2) multiple cleavages after aspartic acid residues at pH <4.5, which finally result in AP-LSAM degradation. Activation likely proceeds via a short-lived intermediate state that has the α6-helix selectively removed but the LSAM domain still bound to the AEP domain (indicated by transparent coloring). Whereas all AtLEGβ activation states show highest conformational stability at intermediate pH, two-chain (pro)AtLEGγ is stable at neutral to slightly acidic pH, and monomeric two-chain AtLEGγ, as well as the AEP domain, are most stable at acidic pH.

Dimerization is a critical regulatory event for caspase-like proteins. In the case of the apoptotic caspases, dimerization is mediated primarily by the β6 strand on the catalytic domain and is associated with structural rearrangements that render the caspase active (Fig. S1). Similarly, dimerization was also observed in plant legumains. The crystal structures of OaAEP1 (PDB entry 5hoi) and AtLEGγ (5nij) both showed a dimer state that was mediated by the α6 and α7 helices on the LSAM domain. However, in these cases dimerization was not associated with activation but rather with inactivation. Under conditions where dimerization is maintained, such as high protein concentration, the proenzyme will not autoprocess to the active AEP form. Additionally, there is a two-chain intermediate state, which is active to some extent. In this study, we show for the first time that there are isoform-specific differences in the activation and activity regulation of *A. thaliana* legumains. First, we observed that proAtLEGβ is monomeric in solution. In this respect, autocatalytic activation of proAtLEGβ more resembles the mechanism known from mammalian legumain, which also lacks a stable, latency-conferring dimer state (Fig. 10). We should point out, however, that in the crystal we found six equivalent proAtLEGβ dimers per asymmetric unit. Nonetheless, this atypical dimer interaction is transient and short-lived and, hence, could not be observed in solution experiments. Second, we found that the AEP–LSAM interface is rather hydrophobic and not charged in nature. Consequently, the stability profile of AtLEGβ differs from AtLEGγ and mammalian legumains (Fig. 10). Third, AtLEGβ encodes autocatalytic cleavage sites on both ends of the α6-helix (Asn345 and Asp363), which in principle allows the selective removal of the AP, like in mammalian legumain (32). Whereas N-terminal cleavage was observed at pH <6.0, cleavage on the C-terminal end of the α6-helix is restricted to pH <5.0, which is in agreement with the charge requirements of the S1 pocket (Fig. 5*A* and 6*B*). Additionally, at acidic pH the ionic clamp that is linking the N-terminal end of the α6-helix (Arg347) to the active site (Glu212) will loosen (Fig. 3*C*), which will further facilitate the release of the AP (α6-helix). Therefore, an AEP–LSAM complex might represent a critical intermediate state, which initiates the complete removal of the LSAM domain by proteolytic degradation and/or conformational destabilization. However, as we did not observe a stable AEP–LSAM complex in our experiments, it will only be short lived (Fig. 5 and 10).

These unique characteristics provide a new regulatory mechanism distinct from that of AtLEGγ. Different oligomerization states will cause AtLEGβ to favor activation at high local concentrations but will favor the latent two-chain state in AtLEGγ. On the other hand, the transient dimers observed in the AtLEGβ crystal might play a role in cooperative substrate processing. Together, these findings suggest that AtLEGβ and γ represent examples of two distinct classes of plant legumains, concerning their physiological function and also with regard to completely different mechanisms of zymogenicity, activation, and stability.

All plant legumains are specific for cleaving after P1-Asn. However, we could show that subtle differences in the nonprime substrate binding sites translate into pronounced kinetic differences. Consequently, different legumain isoforms will feature kinetically driven substrate preferences, which may be modulated by the amount and time of substrate availability (Fig. 7*A*). We provide evidence that the c381-loop can encode such kinetic differences. The corresponding sequences and conformations differ significantly in plant legumains, making it the single most variable region within the plant legumain catalytic domain. Differences in substrate affinity (*K_m_*) can be kinetically assayed using specific substrates. PICS assays with proteome-derived peptide libraries are typically insensitive to such differences because of the mixed and unknown concentration of individual peptide substrates. However, if the substrate affinity is extremely different, such preferences can become apparent. Indeed, using time series experiments, we show that P1-Asp is a low-affinity legumain substrate at increasing pH values. Presenting a P1-Asp consequently may serve as a strategy to kinetically regulate substrate turnover, *i.e.* to release a certain cleavage product in a slow and pH-controlled manner. An example includes the autocatalytic activation of proAtLEGβ, which critically depends on cleavage after Asp residues on the LSAM domain and which is thereby restricted to low pH. Together, this indicates that the differences in the c381-loop among the plant legumains will have an impact on cleavage kinetics rather than on sequence specificity. In line with these observations, we found mostly overlapping AtLEGβ and γ cleavage sites in protein substrates *in vitro*.

Previously, the c381-loop was described as a marker of ligase activity (28). More precisely, a deletion in that region was associated with an increase in ligase activity. However, both AtLEGβ and γ encode relatively long c381-loops, yet both are active ligases. Furthermore, we could show that not only the sequence but also the conformation of this loop can be quite different, although it might be similar in length (Fig. S4). Therefore, we suggest that the c381-loop is primarily a determinant of protease activity. Because protease and ligase activities are inversely coupled, the c381-loop may be an indirect marker of ligase activity. If the affinity of the nonprime (protease) substrate is low, the affinity of the prime-side ligase substrate might be relatively high in comparison. Such a situation favors transpeptidation over substrate hydrolysis. Furthermore, low affinity of nonprime substrates may also result in less recleavage of cyclic products and thereby again indirectly favor ligation. This hypothesis also fits with our observation that AtLEGβ, which has a nonprime binding site optimized for low-affinity binding, is a ligase with broad substrate specificity.

In general, we found that SFTI-derived peptides harboring Asp at P1 position are better ligase substrates resulting in most efficient formation of cyclic product. This observation fits with the notion that poor (high *K_m_*) nonprime substrates are more likely to find a prime ligase substrate at the active site, which in turn excludes the catalytic water molecule from the active site. In concert, the poor nonprime substrate affinity should favor aminolysis of prime substrate over hydrolysis by the catalytic water that is excluded from the active site. For P1-Asp substrates this is particularly true at near-neutral pH, where ligation is favored. Additionally, the residence time of the ligation product is very short, making recleavage of the cyclic product unfavorable and consequently indirectly stabilizing the cyclic product. However, l-SFTI, which lacks P1′ and P2′ residues, resulted in less formation of cyclic product, indicating that P1-Asp will only be tolerated as a substrate at near-neutral pH if coupled to prime side amino acids. P1-Asn as a free C-terminal end worked better, probably because Asn is, in general, a better *K_m_* substrate at pH 6.0. The *K_m_* likely will also be influenced by prime side residues. As a result, a substrate with P1-Asp linked to prime side amino acids will have a critically superior (lower) *K_m_* than C-terminally free Asp, giving the P1-Asp substrate the possibility for binding and transpeptidation. Looking at the prime substrate binding sites, we found that AtLEGβ and γ encode nearly identical substrate binding sites. Taken together, differences in ligation efficiency between AtLEGβ and γ might be explained by their different nonprime substrate binding sites optimized for low- and high-affinity binding, respectively.

In addition to the marker of ligase activity, Cys247 (*O. affinis* numbering) was identified as a gatekeeper residue for ligase activity (20). Mutation to Ala247 resulted in an enzyme with superior ligase activity. Because all AtLEG isoforms harbor a glycine at the equivalent position (Gly241, AtLEGβ numbering), this residue cannot explain the observed isoform specific differences in ligase activity. Similarly, the sequence motif Gly171-Pro172 (AtLEGβ numbering; Fig. S1), which is located close to the S1′ pocket and was recently found to be beneficial for ligase activity, is conserved in both *A. thaliana* legumains (30). However, directly next to Gly241 is Tyr240, which is a critical part of the nonprime binding site (S2–S3) and which is different in AtLEGγ (Trp248). Based on this observation and the differences in the nearby c381-loop, we suggest that it is rather the overall architecture of the nonprime substrate binding sites that affects substrate affinity and might positively affect ligase activity.

The ability of AtLEGβ to join free ends is also interesting from a biotechnological point of view, as it will allow us to link targets without the necessity of introducing artificial cleavage sites, still with the prerequisite of P1-Asn or Asp. Furthermore, it also highlights that joining free termini is a general feature encoded in selected plant legumain isoforms. Given that all plants express a variety of different legumain isoforms, it is very likely that there is an AtLEGβ-like enzyme present in every plant.

Previously, we could show that the two-chain state observed in AtLEGγ is especially interesting with regard to ligase activity, as it is stable at neutral pH environments where ligase activity is favored. Because two-chain AtLEGβ has the same pH stability profile as active AEP, with a pH stability optimum at 5.0, two-chain AtLEGβ will most likely not be a superior ligase. However, it may implement differences in substrate specificity and catalytic efficacy. Indeed, we could previously demonstrate that human two-chain legumain with the C-terminal LSAM domain still present exhibits carboxyl-peptidase activity rather than endopeptidase activity (32). The carboxypeptidase activity is structurally encoded by LSAM-derived arginine residues, which anchor the carboxyl terminus at the primed recognition site. Interestingly, we observed a slight preference for aspartate and glutamate residues in the P1′ position of protein substrates (Fig. 8*B*) together with a relative depletion of basic residues (K and R), which could similarly indicate carboxypeptidase activity of two-chain AtLEGβ. However, this observation has to be taken with some caution, as the relative increase in specificity for Asp and Glu at the P1′ position was low. Finding out whether or not two-chain AtLEGβ indeed harbors carboxy-peptidase activity will require further experiments and may be the subject of future studies.

## Experimental procedures

### Protein preparation

The *Arabidopsis thaliana* vacuolar processing enzyme (VPE, legumain) isoform β (AtLEGβ) full-length clone U12200 (locus AT1G62710) was obtained from the Arabidopsis Biological Resource Center (ABRC). Using this as a template, we subcloned an N-truncated variant missing the N-terminal signal sequences into the pLEXSY-sat2 (Jena Bioscience, Germany) vector using PCR amplification and XbaI and NotI restriction enzymes. The final expression construct carried an N-terminal signal sequence for secretory expression in the LEXSY supernatant and an N-terminal His_6_ tag followed by a TEV recognition site. Furthermore, we prepared a C211A dead mutant using the round-the-horn site-directed mutagenesis technique, which is based on the inverse PCR method (42). Primers were designed that allowed the amplification of the cyclic plasmid template, harboring the proAtLEGβ WT insert, to a linear full-length PCR product carrying the desired mutation on one end of the PCR product. Following gel extraction of the PCR product and blunt-end ligation, an intact plasmid carrying the desired mutation was generated and transformed into *E. coli* Xl2(blue) cells. The C211A mutant was used for crystallization experiments. Correctness of all constructs was confirmed by DNA sequencing. The generated expression constructs were stably transfected into the LEXSY P10 host strain, and stable cell lines were grown as described previously (21). Protein expression and purification was performed as described elsewhere (21, 31). The final proAtLEGβ protein was stored in a buffer composed of 20 mm Hepes, pH 7.0, and 50 mm NaCl. ProAtLEGγ was prepared by following the same protocol.

### Crystallization, data collection, and refinement

Initial screening was performed using the sitting-drop vapor-diffusion method utilizing a Hydra II Plus one liquid-handling system. Crystals of proAtLEGβ were obtained in a condition composed of 0.5 M ammonium sulfate, 1 M lithium sulfate, and 0.1 M trisodium citrate. Crystals grew within 2 weeks at a protein concentration of 10 mg/ml. To prevent autocatalytic activation, we used a C211A dead mutant. Following preincubation in a cryoprotectant solution containing 0.8 M ammonium sulfate, 1.5 M lithium sulfate, 0.1 M trisodium citrate, and 10% sucrose, crystals were flash frozen in liquid nitrogen and subjected to X-ray measurements. A high-resolution data set was collected at the ESRF on beamline ID30B. The beamline was equipped with a Pilatus 6M detector. Data collection was performed at a wavelength of 0.94 Å, 0.037 s exposure time, and 15.3% transmission. 1000 images were collected at an oscillation range of 0.1° and 100 K. Diffraction images were processed using xds and scala from the CCP4 program suite (43, 44). An initial model could be obtained by molecular replacement using PHASER (45), using the crystal structure of two-chain AtLEGγ combined with the sequence of proAtLEGβ. Following iterative cycles of model building in coot (46) and refinement in phenix (47), a final model was obtained and coordinates and structure factors were deposited to the PDB under the accession code 6YSA.

Electrostatic surface potentials were created with APBS (48) after assigning charges at pH 7.0 using Pdb2pqr (49). Surface potentials were contoured at ±5 kT/e.

### Autoactivation

To test the pH dependence of autoactivation of proAtLEGβ, we incubated it at a concentration of 0.4 mg/ml in buffer composed of 100 mm buffer substance (pH 3.5–6.0, citric acid; pH 6.5, MES; pH 7.0, Hepes), 100 mm NaCl, and 2 mm DTT for 1 h at 25 °C. Reactions were stopped by the addition of 10 mm MMTS (S-methyl methane thiosulfonate; Sigma-Aldrich) before subjecting them to SDS-PAGE.

To generate active AtLEGβ on a large scale, we incubated the proenzyme in a buffer composed of 100 mm citric acid, pH 4.0, 100 mm NaCl, and 2 mm DTT at 25 °C for 1 h. Completion of autoactivation was analyzed by SDS-PAGE. Active AtLEGβ was buffer exchanged using a NAP column (GE Healthcare) preequilibrated in a buffer composed of 20 mm citric acid, pH 4.0, and 50 mm NaCl. Active AtLEGγ was prepared by following the protocol described in reference 21.

### Enzymatic activity assays

The enzymatic activity of active AtLEGβ was investigated using the peptidic Z-Ala-Ala-Asn-7-amino-4-methylcoumarin (Z-AAN-AMC; Bachem) and Z-Val-Ala-Asn-AMC (VAN-AMC) substrates. Activity was measured in assay buffer composed of 100 mm citric acid, pH 5.5, 100 mm NaCl, 2 mm DTT, and 100 μm of the respective substrate at 25 °C after adding the enzyme at 60 nm concentration. Assays were carried out in an infinite M200 plate reader (Tecan). Increase in fluorescence was measured at 460 nm upon excitation at 380 nm. *K_m_* values were determined upon incubation of AtLEGβ or γ with serial dilutions of the AAN-AMC substrate in assay buffer. Kinetic data were processed using GraphPad, and *K_m_* values were calculated using implemented algorithms.

### Characterization of oligomerization state

To test the oligomerization state of proAtLEGβ, 200 µl of sample was loaded on a S200 10/300 GL column (GE Healthcare) equilibrated in a buffer composed of 20 mm Hepes, pH 7.5, and 100 mm NaCl. To test the oligomerization state of pH 5.0-activated AtLEGβ, we loaded it on an S200 column preequilibrated in buffer composed of 20 mm citric acid, pH 5.0, and 100 mm NaCl. BSA served as a size standard.

### Determination of melting temperatures

To access the thermal stability of proAtLEGβ and pH 4.0-activated AtLEGβ, we used the Thermofluor method. Experiments were setup as described previously (50). The investigated assay buffers were composed of 100 mm buffer substance (pH 4.0, 5.0, 6.0, citric acid; pH 7.0, Hepes) and 100 mm NaCl. Fluorescence data were analyzed as described elsewhere (51).

### Western blotting

Protein samples to be analyzed were separated on an SDS-PAGE gel. Subsequently, proteins were blotted onto an Amersham Biosciences Protran 0.45 NC membrane (GE Healthcare) using a Trans-Blot SD semi-dry transfer cell (Bio-Rad). The membrane was blocked with 1× TBST supplemented with 5% (w/v) nonfat dry milk. Subsequently, the membrane was incubated with 5% milk-TBST supplemented with 1:10,000 (v/v) anti-His-HRP antibody (ROTH). Chemiluminescent detection of His-tagged protein was performed by using the Amersham Biosciences ECL prime Western blotting detection reagent (GE Healthcare) together with an Odyssey Fc imaging system (Li-Cor).

### Substrate specificity profiling

To test the substrate specificity of AtLEGβ and γ, we carried out proteomic identification of protease cleavage sites (PICS) assays using peptide libraries generated from *Escherichia coli* Bl21 cells (37, 38). The peptide library was prepared as described previously (52). The proteome (2.2 mg/ml) was digested with trypsin at a ratio of 1:100 in 100 mm Hepes, pH 7.5, overnight at 37 °C. The peptide library (2 mg/ml) was incubated with AtLEG proteases (10 µg/ml) in assay buffer composed of 50 mm buffer substance (pH 4.0 and 5.5, citric acid; pH 6.5, MES) and 100 mm NaCl at 25 °C. Samples were taken after 1 h and 18 h of incubation. Protease treated samples were stable isotope labeled with 20 mm heavy formaldehyde (^13^CD_2_O) and 20 mm sodium cyanoborohydride and control reactions with 20 mm light formaldehyde (CH_2_O) and 20 mm sodium cyanoborohydride for 2 h and quenched with 100 mm Tris, pH 8.0, for 1 h. Protease-treated and control samples were mixed and purified using C_18_ StageTips.

### Substrate specificity profiling of AtLEGβ and γ using intact A. thaliana leaf proteome

*A. thaliana* VPE quadruple mutant (VPE0 [53]) was obtained from the Nottingham Arabidopsis Stock center (accession N67918). Leaves were harvested from 8-week-old plants grown on soil under short-day conditions (9 h/15 h photoperiod, 22 °C/18 °C, 120 µmol photons m^−2^ s^−1^). The harvested leaves were homogenized with a Polytron PT-2500 homogenizer (Kinematica, Luzern, Switzerland) in extraction buffer containing 0.05 M MES, pH 6.0, 0.15 M NaCl, 10% (w/v) sucrose, 0.01 M DTT, and HALT protease inhibitor mixture (ThermoFisher, Dreieich, Germany) on ice. The lysate was then filtered through Miracloth (Merck, Darmstadt, Germany), followed by centrifugation at 4000 × *g* at 4 °C for 5 min. The protein concentration was determined by the Bradford assay using BSA as a reference.

Recombinant AtLEGβ, recombinant AtLEGγ, or buffer control were added to the isolated proteome at a protease-to-proteome (1 mg) ratio of 1:100 (w/w) in the extraction buffer and incubated in parallel at 25 °C for 3 h. The reactions were terminated by addition of 50 μm caspase-1 inhibitor (YVAD-cmk, Bachem, Switzerland). The reaction mixtures were purified by chloroform-methanol precipitation (54) and resuspended in 6 M GuaHCl, 0.1 M HEPES, pH 7.5. The protein concentrations were determined using the BCA assay (ThermoFisher, Dreieich, Germany). The digested proteomes were reduced with 5 mm DTT at 56 °C for 30 min followed by alkylation with 15 mm iodoacetamide for 30 min at 25 °C and quenched by addition of 15 mm DTT for 15 min. The three samples were differentially dimethyl labeled with 20 mm light formaldehyde (^12^CH_2_O) and 20 mm sodium cyanoborohydride (light label), 20 mm medium formaldehyde (^12^CD_2_O) and 20 mm sodium cyanoborohydride (medium label), or 20 mm heavy formaldehyde (^13^CD_2_O) and 20 mm sodium cyanoborodeuteride. After 16 h of incubation at 37 °C, the same amounts of fresh reagents were added and incubated for another 2 h. The reactions were quenched with 0.1 M Tris (final concentration) at pH 7.4 and 37 °C for 1 h. Equal amounts of protein were pooled, purified by chloroform-methanol precipitation, and resuspended in 0.1 M HEPES, pH 7.4. The sample was then digested with trypsin in a 1:100 protease:protein ratio (SERVA Electrophoresis, Heidelberg, Germany) at 37 °C for 16 h. Enrichment of N-terminal peptides was performed according to the HUNTER method (40). In brief, trypsin-digested sample was tagged with undecanal at a ratio of 50:1 (w/w) in 40% ethanol supplemented with 20 mm sodium cyanoborohydride at 50 °C for 45 min. An additional 20 mm sodium cyanoborohydride was added for another 45 min under the same condition. The reaction was then acidified with a final concentration of 1% TFA and centrifuged at 21,000 × *g* for 5 min. Next, the supernatant was injected through a pre-activated HR-X (M) cartridge (Macherey-Nagel, Düren, Germany). The flowthrough containing N-terminal peptides was collected. Remaining N-terminal peptides on the HR-X (M) cartridge were eluted with 40% ethanol containing 0.1% TFA, pooled with the first eluate and subsequently evaporated in the SpeedVac to a small volume suitable for C_18_ StageTip purification prior to mass spectrometric analysis. The assays were performed in three biological triplicates.

### MS data acquisition

Samples were analyzed on a two-column nano-HPLC setup (Ultimate 3000 nano-RSLC system with Acclaim PepMap 100 C_18_, ID of 75 μm, particle size of 3 μm, a trap column of 2-cm length and analytical column of 50 cm length, ThermoFisher) with a binary gradient from 5–32.5% B for 80 min (A, H_2_O + 0.1% FA; B, ACN + 0.1% FA) and a total runtime of 2 h per sample, coupled to a high-resolution Q-TOF mass spectrometer (Impact II, Bruker) as described previously (55). Data were acquired with the Bruker HyStar Software (v3.2, Bruker Daltonics) in line-mode in a mass range from 200–1500 *m*/*z* at an acquisition rate of 4 Hz. The top 17 most intense ions were selected for fragmentation with dynamic exclusion of previously selected precursors for the next 30 s, unless intensity increased 3-fold compared with the previous precursor spectrum. Intensity-dependent fragmentation spectra were acquired between 5 Hz for low-intensity precursor ions (>500 cts) and 20 Hz for high-intensity (>25,000 cts) spectra. Fragment spectra were averaged from t-stepped parameters, with 50% of the acquisition time manner with split parameters: 61-µs transfer time, 7 eV collision energy, and a collision RF of 1500 Vpp, followed by 100-µs transfer time, 9 eV collision energy, and a collision RF of 1800 Vpp

### MS data analysis

Acquired mass spectra were matched to peptide sequences at an FDR of 0.01 using MaxQuant (56) v.1.6.0.16 using standard Bruker QToF instrument settings. For PICS experiments, the UniProt *E. coli* K12 proteome database (downloaded November 2015, 4313 entries) with appended common contaminants was used. Search parameters considered semispecific tryptic peptides, light (+28.031300) and heavy (+36.075670) dimethyl labeling at peptide N-termini or Lys side chain amines, and Cys carbamidomethylation as fixed and Met oxidation as variable modifications. Identified peptides that showed at least a 4-fold increase in intensity after protease treatment compared with the control treatment or were exclusively present in the protease-treated condition were considered putative cleavage products. An in-house Perl script was used to remove putative library peptides (trypsin specificity on both sides of the identified peptide) and to reconstruct the full cleavage windows from the identified cleavage products as described previously (38) and visualized as IceLogos using software version 1.3.8 (57).

For HUNTER experiments, the *A. thaliana* UniProt proteome database (downloaded December 2018, 41,592 entries) with appended list of common laboratory contaminants was used for searches that considered C-terminal cleavage by ArgC as digestion enzyme. Further search parameters included isotope labeling by light (+28.031300), medium (+32.056407), or heavy (+36.075670) dimethylation of peptide N-termini or Lys residues, Cys carbamidomethylation as fixed and Met oxidation, N-terminal acetylation (+42.010565), or N-terminal pyroGlu formation from Glu (−18.010565) or Gln (−17.026549) as variable modifications. Further statistical data analysis, filtering and annotation were performed with the Perl script MANTI.pl 3.9.7 (https://manti.sourceforge.io).

### Peptide cyclization assay

SFTI-derived peptides were synthesized and analyzed as described previously (29). Subsequently, cyclization experiments were carried out using 500 μm of the respective linear peptide and 0.5 μm AtLEGβ in a buffer composed of 100 mm NaCl and 50 mm Tris, Bis-Tris, citric acid, pH 4.0 or pH 6.0. Reactions were incubated at 30 °C for 12 h. Subsequently, the reactions were desalted using ZipTip C_18_ tips (Merck Millipore) and analyzed by MALDI-TOF-MS (Autoflex, Bruker Daltonics, matrix, α-cyano-4-hydroxycin-namic acid).

## Data availability

The coordinates and structure factors presented in this paper have been deposited with the Protein Data Bank (PDB) with the accession code 6YSA. MS data have been deposited with the PRIDE (58) repository with the accession codes PXD019220 for the PICS data set and PXD019276 for the HUNTER N-terminome data set. All remaining data are contained within the article.

## Supplementary Material

Supporting Information
