# Supplementary material for: Structural and functional studies of Arabidopsis thaliana legumain beta reveal isoform specific mechanisms of activation and substrate recognition
Source: J Biol Chem. 2020 Jul 21;295(37):13047–64. doi: 10.1074/jbc.RA120.014478 (PMC7489914; doi:10.1074/jbc.RA120.014478)
Supplement: Supporting Information [file supp_295_37_13047__index.html]

Structural and functional studies of Arabidopsis thaliana legumain beta reveal isoform specific mechanisms of activation and substrate recognition — Structure and Function of AtLEGβ — Structural and functional studies of Arabidopsis thaliana legumain beta reveal isoform specific mechanisms of activation and substrate recognition — Structure and function of AtLEGβ — Supporting Information 

# Structural and functional studies of *Arabidopsis thaliana* legumain beta reveal isoform specific mechanisms of activation and substrate recognition

## Supporting Information

- Supporting Information (to be published online) - Supporting Information
- Supporting Information (to be published online) - Supplementary Table S1
