# Supplementary material for: Structural and functional studies of Arabidopsis thaliana legumain beta reveal isoform specific mechanisms of activation and substrate recognition
Source: J Biol Chem. 2020 Jul 21;295(37):13047–64. doi: 10.1074/jbc.RA120.014478 (PMC7489914; doi:10.1074/jbc.RA120.014478)
Supplement: Supporting Information [file supp_RA120.014478_161009_2_supp_567284_qds9t6.pdf]

SUPPORTING INFORMATION

Structural and functional studies of *Arabidopsis thaliana* legumain beta reveal isoform specific mechanisms of activation and substrate recognition

Authors:
Elfriede Dall[†*#], Florian B. Zauner[†*], Wai Tuck Soh[†], Fatih Demir[§], Sven O. Dahms[†], Chiara Cabrele[†], Pitter F. Huesgen[§‖⊥], Hans Brandstetter[†#].

Affiliation:
[†]Department of Biosciences, University of Salzburg, 5020 Salzburg, Austria.
[§]Central Institute for Engineering, Electronics and Analytics, ZEA-3, Forschungszentrum Jülich, 52428 Jülich, Germany.
[‖]CECAD, Medical Faculty and University Hospital, University of Cologne, 50931 Cologne, Germany
[⊥]Institute for Biochemistry, Faculty of Mathematics and Natural Sciences, University of Cologne, 50674 Cologne, Germany
*These authors contributed equally to this work.

[#]Corresponding authors:
Elfriede Dall: elfriede.dall@sbg.ac.at
Hans Brandstetter: hans.brandstetter@sbg.ac.at

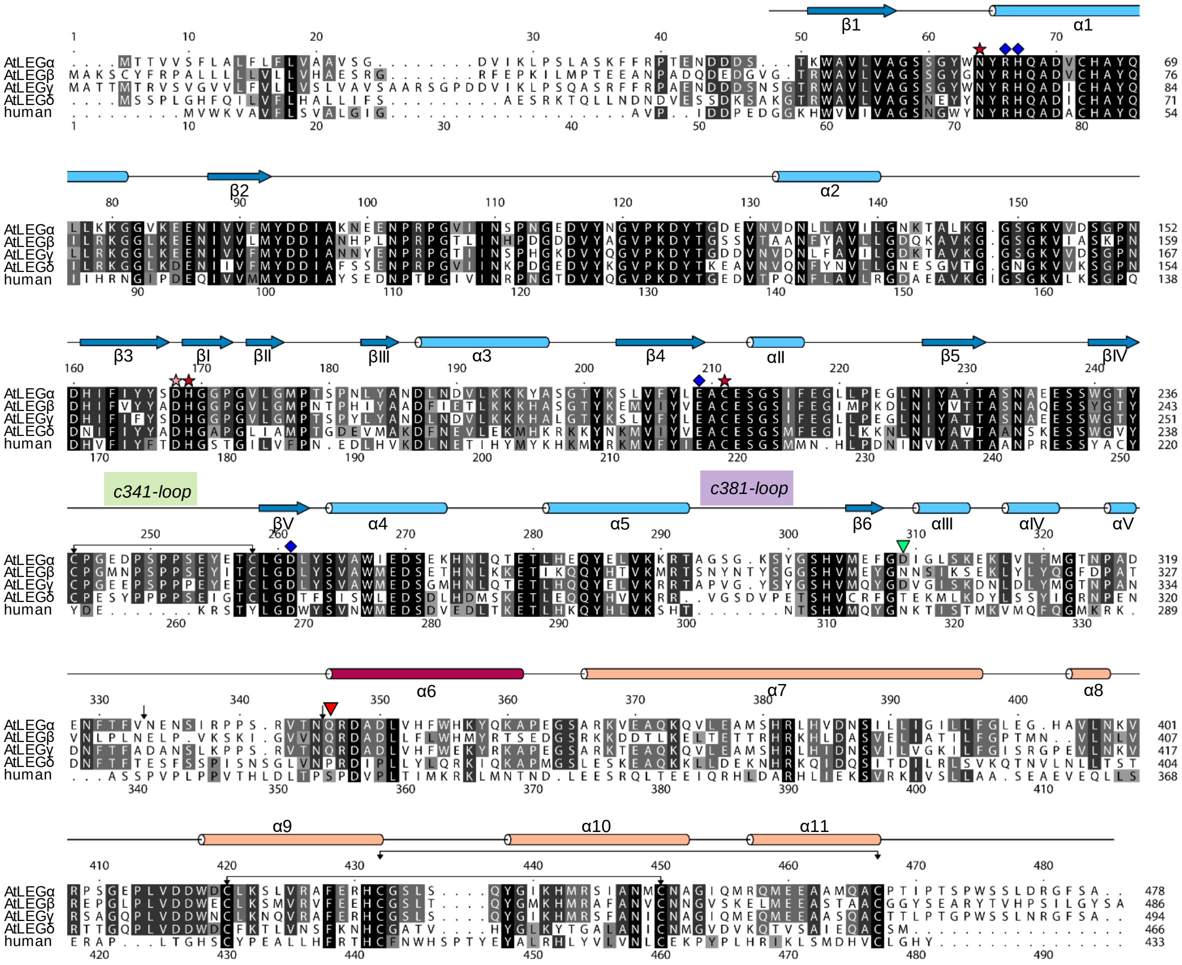

Supporting Figure S1. Sequence alignment of *Arabidopsis thaliana* (AtLEGα: P49047, AtLEGβ: Q39044, AtLEGγ: 5nij, AtLEGδ: Q9LJX8) and human legumain (4fgu). Arrows indicate autocatalytic cleavage sites, red stars: catalytic residues, green triangle: glycosylation site, blue diamonds: residues forming the S1-specificity pocket. Secondary structure elements are based on the crystal structure of proAtLEGβ. The numbering on top of the alignment is based on the AtLEGβ sequence, and the numbering below the alignment on the AtLEGγ sequence. The alignment was created with Clustal W (1) and modified with Aline (2).

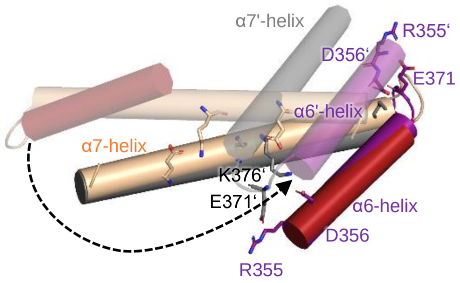

Supporting Figure S2. Superposition of 4-helix bundles observed in proAtLEGβ (pdb entry 6ysa) and 2-chain AtLEGγ (pdb entry 5nij).

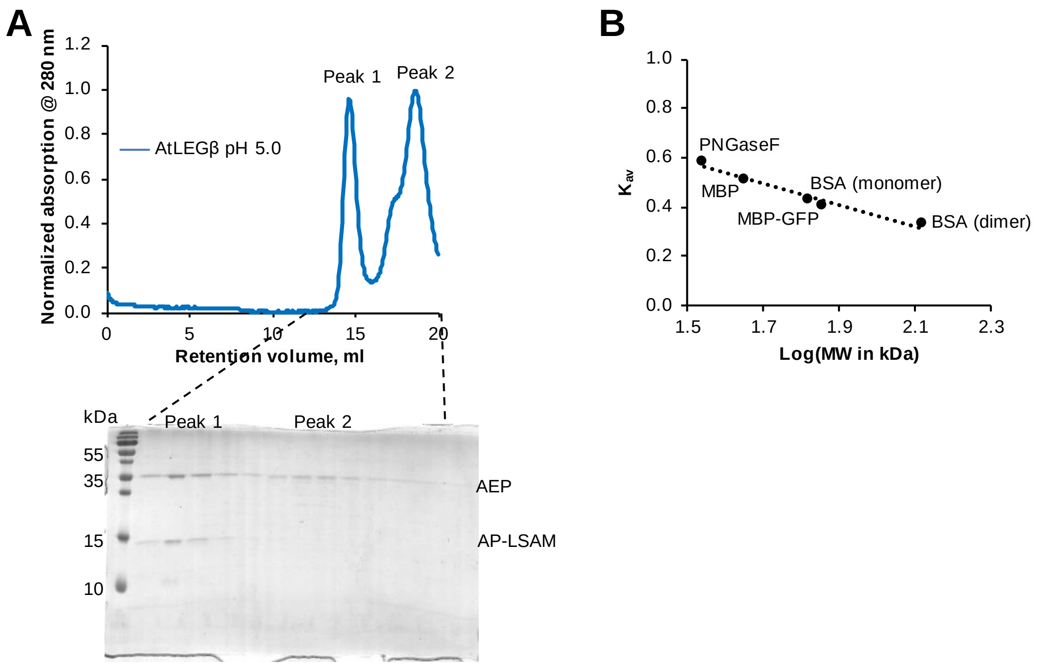

Supporting Figure S3. AtLEGβ forms a 2-chain state upon activation at pH 5.0. **A.** Two-chain AtLEGβ migrates at the expected size of a monomer. Co-migration of AEP and LSAM domains in 2-chain AtLEGβ was evidenced by SDS-PAGE. **B.** A calibration curve of the S200 SEC column used in the experiment described in panel A was prepared using 4 different standard proteins, following the manufacturer's instructions (3). K_{av} was calculated using the equation $K_{av} = (v_e\text{-}v_0)/(v_c\text{-}v_0)$ where v_e is the elution volume of the standard protein, v_0 is the void volume and v_c is the column volume (MBP: maltose binding protein; GFP: green fluorescent protein). Linear fitting resulted in a K_{av} of 1.87 of the proAtLEGβ peak, corresponding to a theoretical molecular weight of 72 – 66 kDa, which is in rough agreement with the monomer state of proAtLEGβ.

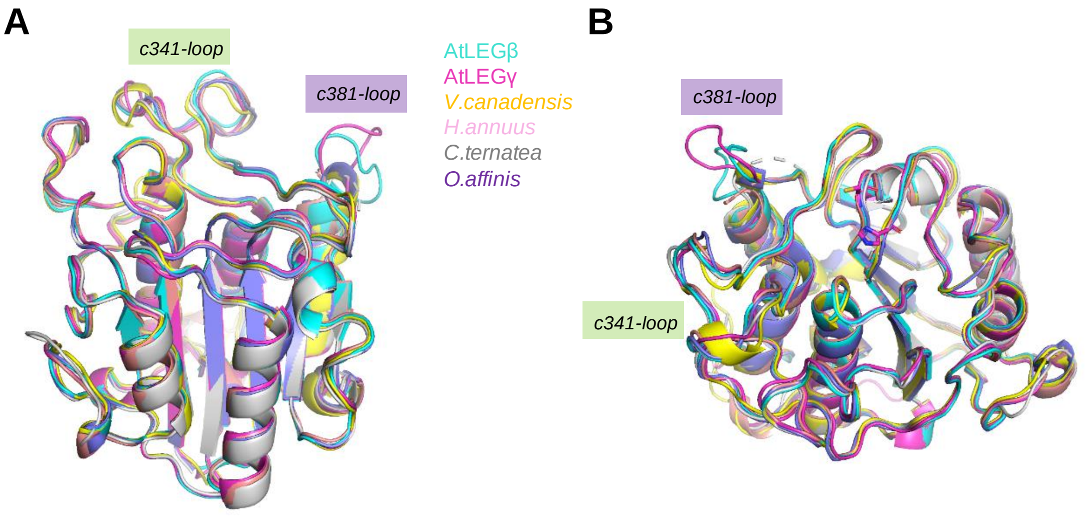

Supporting Figure S4. A. Superposition of plant legumain structures. AtLEGβ (pdb entry 6ysa), AtLEGγ (pdb entry 5obt), *V.canadensis* (pdb entry 5zbi), *H. annuus* (pdb entry 6azt), *C. ternatea* (pdb entry 6dhi), *O. affinis* (pdb entry 5h0i). The view in **B.** is rotated by 90° along the y-axis and 90° along the z-axis.

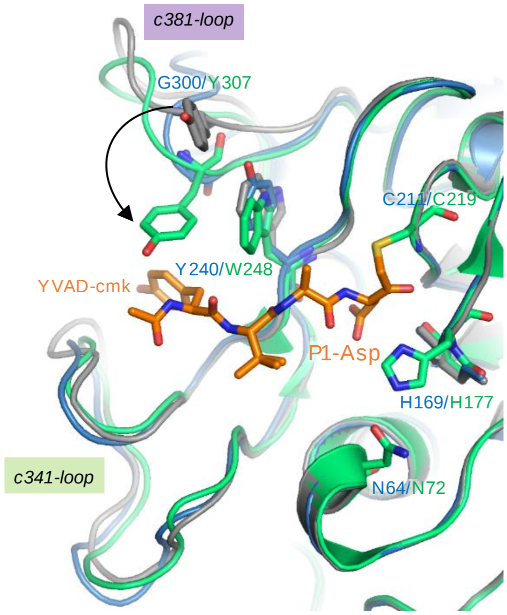

Supporting Figure S5. The active site of proAtLEGβ most closely resembles the active state of AtLEGγ. Superposition of the crystal structures of proAtLEGβ (blue), AtLEGγ (5obt, green) and zymogenic two-chain AtLEGγ (5nij, grey). The covalent YVAD-cmk inhibitor that labeling the active site of AtLEGγ (5obt) is shown in orange sticks.

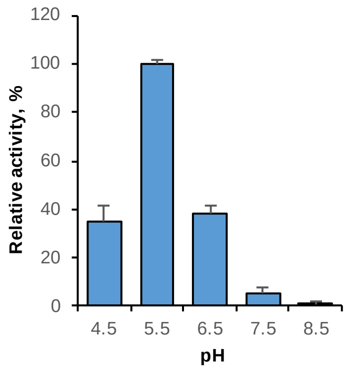

Supporting Figure S6. AtLEGβ shows decreasing activity near neutral pH. Activity was measured using the Z-AAN-AMC fluorogenic substrate at indicated pH values. Activity was normalized to the highest activity, which was measured at pH 5.5.

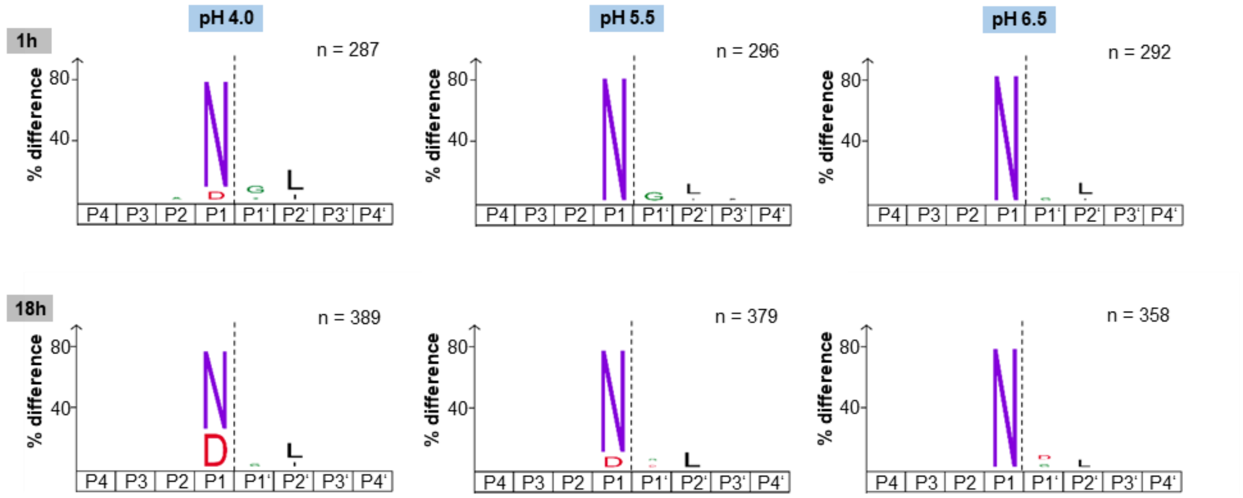

Supporting Figure S7. AtLEGγ has a pH and time dependent substrate specificity. Cleavage site specificity was determined by the PICS assay, using peptides generated by tryptic digest of an *E.coli* proteome as substrate library. iceLogos visualize the substrate preference surrounding the cleavage sites ($p = 0.05$). Digestion was performed at indicated pH values and times. Number of non-redundant cleavage sites used to generate the iceLogos are indicated.

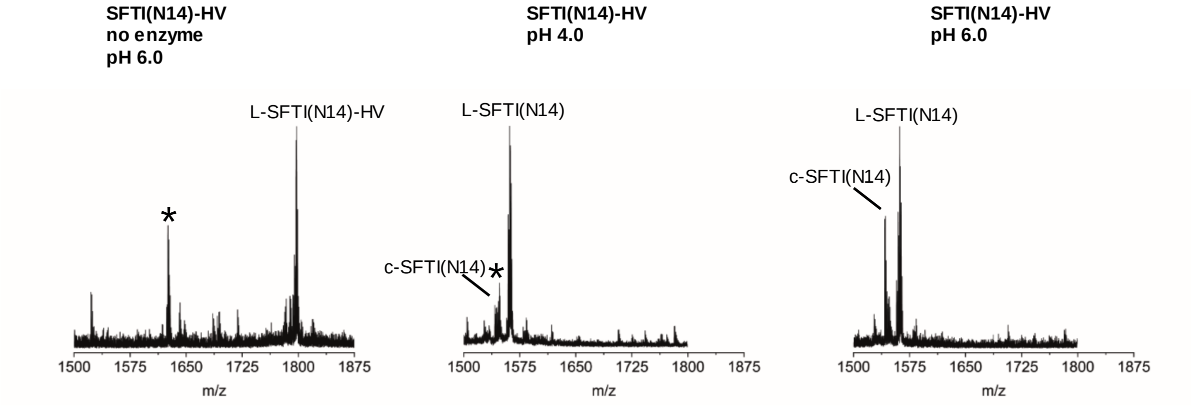

Supporting Figure S8. Cyclisation of SFTI-derived peptides by AtLEGβ is pH-dependent. Reactions were carried out at indicated pH values. An asterisk labels an unidentified species.

References

1. Thompson, J. D., Higgins, D. G., and Gibson, T. J. (1994) CLUSTAL W: improving the sensitivity of progressive multiple sequence alignment through sequence weighting, position-specific gap penalties and weight matrix choice. *Nucleic Acids Res* **22**, 4673-4680
2. Bond, C. S., and Schuttelkopf, A. W. (2009) ALINE: a WYSIWYG protein-sequence alignment editor for publication-quality alignments. *Acta Crystallogr D Biol Crystallogr* **65**, 510-512
3. GE Healthcare (2009) Gel Filtration Clibration Kits. *Product booklet*
